# Revisiting eicosapentaenoic acid and docosahexaenoic acid recommendations for healthy Siberian Huskies at maintenance: greater dietary EPA and DHA increase plasma lipid incorporation and reduce α-tocopherol concentrations

**DOI:** 10.1093/jas/skag257

**Published:** 2026-08-13

**Authors:** Benjamin D Fox, Fumiko Imai, Sydney Banton, Alexandra Rankovic, Sarah MacDonald Murray, Lindsey Rummell, David W L Ma, Lyn M Hillyer, Jennifer Saunders-Blades, Janelle Kelly, James R Templeman, Lindsay E Robinson, Anna K Shoveller

**Affiliations:** Department of Animal Biosciences, University of Guelph, Guelph, ON N1G 2W1, Canada; Department of Animal Biosciences, University of Guelph, Guelph, ON N1G 2W1, Canada; Department of Animal Biosciences, University of Guelph, Guelph, ON N1G 2W1, Canada; Department of Animal Biosciences, University of Guelph, Guelph, ON N1G 2W1, Canada; Department of Animal Biosciences, University of Guelph, Guelph, ON N1G 2W1, Canada; Department of Human Health and Nutritional Sciences, University of Guelph, Guelph, ON N1G 2W1, Canada; Department of Human Health and Nutritional Sciences, University of Guelph, Guelph, ON N1G 2W1, Canada; Department of Human Health and Nutritional Sciences, University of Guelph, Guelph, ON N1G 2W1, Canada; Champion Petfoods Holding Inc., Edmonton, AB T6X 0P8, Canada; Champion Petfoods Holding Inc., Edmonton, AB T6X 0P8, Canada; Primal Pet Foods, Primal Pet Group, Fairfield, CA 94634, United States; Department of Human Health and Nutritional Sciences, University of Guelph, Guelph, ON N1G 2W1, Canada; Department of Animal Biosciences, University of Guelph, Guelph, ON N1G 2W1, Canada

**Keywords:** antioxidant status, canine nutrition, docosahexaenoic acid, dose-response, eicosapentaenoic acid, serum lipids

## Abstract

Recent evidence suggests that higher-than-recommended intakes of long-chain n-3 polyunsaturated fatty acids (PUFA), specifically eicosapentaenoic acid (EPA) and docosahexaenoic acid (DHA), are both safe and effective in supporting health conditions such as inflammatory diseases in adult humans. For adult dogs at maintenance, the National Research Council (NRC 2006) recommends a safe upper limit of EPA+DHA of 0.37 g/kg body weight (BW)^0.75^, a value based on limited evidence. This study aimed to investigate how low, moderate and high dietary intakes of EPA+DHA affected oxidative balance through α-tocopherol and malondialdehyde (MDA) concentrations, lipid incorporation into circulating phospholipid fractions, and serum cytokines in healthy adult dogs over 8 wk. Twenty-seven client-owned dogs were enrolled and, following a 4-wk diet acclimation phase, were allocated into three groups balanced for age and BW and then randomly assigned to 1 of 3 dosage groups, consisting of 0.03 g/kg BW^0.75^ (LOW) of EPA+DHA, 0.45 g/kg BW^0.75^ (MOD), and 0.71 g/kg BW^0.75^ (HIGH). Fasted blood samples were collected at weeks 0, 4, and 8 to evaluate serum fatty acid (FA) composition within circulating phospholipid fractions, whole blood α-tocopherol, malondialdehyde (MDA), and cytokine concentrations. Data were analyzed using PROC GLIMMIX in SAS, with dog treated as a random effect, week as a repeated measure, and dosage group (DG), week, sex, and their interactions as fixed effects. In serum phosphatidylcholine (PC) and lysophosphatidylcholine (LysoPC) fractions, EPA and DHA incorporation increased from baseline to week 4 across all dosage groups, with no changes observed between weeks 4 and 8. Total FA analysis showed that dietary EPA+DHA increased total n-3 PUFA and reduced total n-6 PUFA, with both the MOD and HIGH being greater than the LOW treatment. Whole blood α-tocopherol concentrations showed a DG × week interaction (*P* = 0.034), with the LOW group (5.82 µg/mL) maintaining higher concentrations at week 8 than the HIGH group (3.52 µg/mL) and not differing from the MOD group (5.00 µg/mL). Overall, increasing dietary EPA+DHA intake in adult dogs enriched circulating and membrane-associated n-3 PUFA pools while maintaining MDA and inflammatory cytokine concentrations despite reduced α-tocopherol concentrations. This suggests that higher EPA+DHA intakes were well tolerated over the 8-wk study period when interpreted alongside antioxidant status.

## Teaser

Feeding dogs higher amounts of omega-3 fatty acids (EPA and DHA) increased blood lipid incorporation but reduced antioxidant status. These findings highlight the ability to improve EPA and DHA status, but also the need to balance EPA and DHA intake with adequate antioxidant support in canine diets.

## Introduction

Long-chain n-3 polyunsaturated fatty acids (PUFA), such as eicosapentaenoic acid (EPA) and docosahexaenoic acid (DHA), are functional fatty acids (FA) that regulate inflammation, immune function, oxidative balance, and cell membrane composition (Calder 2015). Consequently, EPA and DHA supplementation has been investigated extensively in dogs for the nutritional management of osteoarthritis, dermatological disorders, chronic kidney disease, cardiovascular disease, and other inflammatory conditions, with therapeutic doses often exceeding those required for maintenance, as reviewed by Lenox and Bauer (2013).

These documented health benefits support the need to establish a minimum dietary requirement for EPA and DHA while ensuring that intake remains below those that may result in adverse physiological effects. The NRC (2006) guideline provides a recommended allowance (RA) of 0.03 g/kg body weight (BW)^0.75^ and safe upper limit (SUL) of 0.37 g/kg BW^0.75^ for EPA and DHA combined for healthy adult dogs (NRC 2006). Intakes of EPA and DHA above the current SUL have been associated with increased lipid peroxidation, reduced circulating α-tocopherol concentrations, altered platelet function, prolonged bleeding time, and other potential adverse effects, highlighting the importance of defining an appropriate SUL in dogs (Wander et al. 1997; Hall et al. 2003; Lenox and Bauer 2013).

Furthermore, while no minimum requirement has been established for adult dogs at maintenance, the European Pet Food Industry Federation (FEDIAF) currently recommends a minimum dietary concentration of EPA+DHA for growth and reproduction (125 mg/1,000 kcal ME), recognizing the importance of these FA during early development (FEDIAF 2026).

The NRC (2006) SUL is primarily based on a single study in senior Beagles by Wander et al. (1997), which reported dietary EPA+DHA concentrations rather than BW-normalized intakes. To facilitate comparison with the present study, the dietary concentrations reported by Wander et al. (1997) were converted to estimated EPA+DHA intakes (g/kg BW^0.75^) using the reported BW and estimated feed intake. Based on these calculations, the highest dietary treatment from Wander et al. (1997) corresponds to an estimated intake between the MOD and HIGH dosage groups (0.54 g/kg BW^0.75^) evaluated in the present study. Dogs in the highest dosage group exhibited reduced prostaglandin E2 (PGE2) concentrations, indicative of suppressed inflammation, but also increased lipid peroxidation, raising concerns about oxidative stress (Wander et al. 1997). The NRC (2006) SUL was established based on these study findings to minimize potential risks associated with higher EPA and DHA intake. However, it is important to acknowledge the limitations of that study. Although previous studies have established the safety of EPA and DHA supplementation in senior dogs (Wander et al. 1997), further characterization of their incorporation into circulating phospholipid fractions and their relationship with antioxidant and inflammatory biomarkers may improve our understanding of the physiological responses to greater dietary EPA and DHA intakes. Therefore, the objective of the present study was to investigate dose-dependent changes in circulating phospholipid FA composition, α-tocopherol, malondialdehyde (MDA), and cytokine concentrations in a broader age range of dogs and in a different breed from those evaluated previously. To better characterize the incorporation of EPA and DHA into circulating lipid pools, individual phospholipid fractions, including phosphatidylcholine (PC), lysophosphatidylcholine (LysoPC), and sphingomyelin (SM), were evaluated, as these lipid pools exhibit distinct metabolic functions and turnover rates of FA. Previous work has also demonstrated that the biological matrix selected for FA analysis influences the measured EPA and DHA response to supplementation, highlighting the importance of characterizing incorporation within specific circulating lipid fractions (Goffin et al. 2017).

For humans, the National Academy of Medicine recommends a daily intake of 1.6 g and 1.1 g of α-linolenic acid (ALA) for adult males and females, respectively, and up to 10% of this can come from EPA and DHA (Trumbo et al. 2002). However, literature supports the notion that higher doses of n-3 PUFA are safe and may be effective for disease prevention. For example, Bhatt et al. (2019) found that a 4 g daily dose of EPA resulted in a 25% decrease in major cardiovascular events over a 4.6-year follow-up period in men aged 45 and older (Bhatt et al. 2019). Similarly, Yokoyama et al. (2007) reported that supplementation with 1.8 g EPA in 18,645 male and female hypercholesterolemic patients aged up to 75 years led to a 19% reduction in major coronary events over 4.6 years. Based on these human studies, dogs receiving higher-than-recommended doses of EPA and DHA might also gain health benefits without adverse effects. Supporting this notion, a study on osteoarthritic dogs, a population that is not healthy and experiences severe inflammation, evaluated high doses of EPA+DHA (0.21, 0.50, and 0.71 g/kg BW^0.75^). After three months of daily supplementation, dogs receiving the highest dose had improved clinical signs such as reduced lameness and improved range of motion, with no reported adverse effects (Fritsch et al. 2010). Previous work has also demonstrated that dietary EPA and DHA supplementation attenuated lipopolysaccharide (LPS)-stimulated IL-1 and IL-6 production in clinically normal dogs, suggesting that the immunomodulatory effects of long-chain n-3 PUFA may be more readily detected following an inflammatory challenge (LeBlanc et al. 2008). However, the effects of higher dietary EPA and DHA intakes on circulating cytokine concentrations in healthy dogs under non-challenged conditions remain poorly characterized. Nonetheless, studies investigating how higher doses of EPA+DHA affect FA incorporation, oxidative status, and immune responses in otherwise healthy adult dogs are limited. This highlights the need for prospective, hypothesis-driven, dose-response studies to reassess the current NRC (2006) guidelines. It is also important to note that the Association of American Feed Control Officials (AAFCO) does not currently establish a minimum RA or a maximum limit for EPA and DHA in adult dog foods (AAFCO 2026). Therefore, the objective of the current study was to investigate EPA+DHA doses above the current SUL and the effects on serum lipid fractions, α-tocopherol concentrations, and cytokines. We hypothesized that EPA+DHA supplementation would result in a dose-dependent increase in their incorporation into serum phospholipid fractions. At higher doses, we anticipated an increase in lipid peroxidation, accompanied by a decrease in α-tocopherol concentration, reflecting increased oxidative demand and increased utilization of antioxidant α-tocopherol. We also hypothesized that the anti-inflammatory properties of EPA and DHA would result in decreased pro-inflammatory cytokines and increased anti-inflammatory cytokines.

## Materials and methods

### Animals and housing

This study was approved by the University of Guelph’s Animal Care Committee (Animal Utilization Protocol #5229) and was conducted in full compliance with the Canadian Council on Animal Care (CCAC) guidelines and the (Government of Ontario 1990). Twenty-seven client-owned dogs (25 Siberian Huskies and 2 Alaskan Huskies) participated in the study. The group included 13 females (4 intact, 9 spayed) and 14 males (3 intact, 11 neutered) with an age range of 1–12 years (mean ± SD: 6.9 ± 3.2 yr) and a mean BW of 21.5 ± 3.2 kg. The body condition score (BCS) ranged from 4 to 6 on a 9-point scale (mean ± SD: 4.0 ± 3 BCS) (Laflamme 1997). Dogs remained housed with their owner at a private off-site kennel (Rajenn Siberian Huskies, Ayr, Ontario, Canada) and were pair or group-housed in free-run, outdoor kennels ranging from 39 to 896 m², accommodating 2 to 13 dogs per kennel. Each dog had access to dog houses for rest and protection from the weather. All dogs were housed at the same kennel under the same ownership and management throughout the study. The study was completed from November 2024 to January 2025. All dogs underwent a physical examination, complete blood count, serum biochemistry, and medical history review before enrollment to confirm health status prior to and post participation.

### Dietary treatment and study design

Dogs underwent a 4-wk diet acclimation period (week −4 to 0) before the study began. During this time, dogs were transitioned to once-daily feeding at 15:00 h and fed a dry extruded commercial diet (ACANA Wholesome Grains Free-Run Poultry, Champion Petfoods Ltd., Edmonton, Alberta, Canada) containing 750 IU vitamin E/kg on an as-fed basis. At each feeding, dogs were fed individually to ensure accurate feed intake (FI). They were given 1 h to consume their food, after which any offered-refused-treatments (orts) were weighed (Valor™ 2000 digital precision scale, OHAUS Corporation, New Jersey, United States of America) and recorded. During this acclimation period, dogs were initially fed based on historical feeding records to meet maintenance energy requirements and maintain BW. Throughout the study period, dogs were weighed weekly, and FI was adjusted to maintain baseline BW. Body condition score was monitored weekly based on a 9-point scale (Laflamme 1997). All dogs had *ad libitum* access to fresh water, and 200 mL of water was added to their food during daily feeding to increase water intake.

At baseline, dogs were allocated to three groups balanced for age and BW, then randomly assigned to one of three dosage groups (DG), following a randomized complete block design. During the study period, dogs were fed an experimental extruded diet (Champion Petfoods Ltd., Edmonton, Alberta, Canada; nutrient composition detailed in Table S1), which contained low to moderate fat (15.05%), EPA (0.03%), and DHA (0.04%) on a dry matter basis. All analyzed vitamins and minerals were formulated to meet or exceed AAFCO and NRC (2006) recommendations for adult dogs, and aside from vitamin E (1889 IU/kg as fed), they were not considered primary variables of interest in the present study.

Fish oil capsules (OGRAX-3; Biolab Sanus Farmaceutica Ltd., Jandira, Brazil) contained either 500, 1,000, or 1,500 mg fish oil per capsule, providing 130 and 85 mg, 260 and 170 mg, or 600 and 450 mg EPA and DHA per capsule. According to the manufacturer’s certificate of analysis, the fish oil had a peroxide value of 1.0 meq O₂/kg, an anisidine value of 0.8, and an acidity value of 0.1, indicating minimal primary and secondary lipid oxidation before use. Fish oil capsules were stored in their original packaging in a cool, dry location, protected from light, heat, moisture, and air exposure, in accordance with the manufacturer’s recommendations throughout the study to minimize oxidative degradation. Capsules were combined as needed to achieve the target EPA+DHA doses of 0.03 g/kg BW^0.75^ (LOW), 0.45 g/kg BW^0.75^ (MOD), and 0.71 g/kg BW^0.75^ (HIGH). LOW represented the daily RA. The MOD group represented the current NRC (2006) SUL. In contrast, the highest dietary treatment evaluated by Wander et al. (1997) fell between the MOD and HIGH dosage groups used in the present study. The HIGH group represented an EPA+DHA intake exceeding both the current NRC (2006) SUL and the highest estimated intake evaluated by Wander et al. (1997), while remaining within the range of dietary EPA+DHA concentrations previously investigated in canine studies (Hall et al. 2006; Fritsch et al. 2010).

Dogs that readily consumed fish oil capsules were provided with them whole, whereas for those that refused, the capsules were pierced, and the oil was drizzled over the kibble before feeding. For dogs that did not readily consume capsules, the capsules were punctured and emptied onto the food or eaten whole, with residual oil remaining within the capsule, recovered using a micropipette immediately before feeding to ensure the full intended dose was administered and recorded. For all dogs, the fish oil was mixed with half of the daily kibble portion, which was offered first. Once the dogs had fully consumed the initial portion, the remaining portion was provided. This procedure was done to ensure full consumption of the fish oil, which was dosed on a BW basis and remained consistent throughout the trial, and simultaneously allowed calculation of FI. Both the commercial diet and the experimental diet met or exceeded (Association of American Feed Control Officials (AAFCO) 2016) nutrient recommendations for adult dogs at maintenance without the fish oil supplement.

### Blood collection and analysis

Dogs were fasted for 16 h overnight, after which whole blood samples (8 mL) were collected via cephalic venipuncture using a Serum Vacutainer® system (Becton, Dickinson and Company, Franklin Lakes, New Jersey, United States of America) at weeks 0, 4, and 8. After collection, blood tubes were left to clot at room temperature for 30 min before being placed in a cooler box with ice. Samples were then transported to the University of Guelph and centrifuged at 2,500 × g for 15 min at 4 °C using a Beckman J6-MI centrifuge (Beckman Coulter, Indianapolis, Indiana, United States of America). After centrifugation, the serum was aliquoted and stored at −80 °C until analysis of FA composition, α-tocopherol, MDA, and cytokines. α-Tocopherol, MDA, and inflammatory cytokines were selected because they collectively assess antioxidant status, oxidative damage, and systemic inflammatory responses, which are the principal mechanisms by which EPA and DHA are proposed to exert their biological effects.

### Total FA analysis

Serum (50 µL) samples were thawed on ice. To each acid-washed 15-mL glass tube with a polytetrafluoroethylene-lined screw cap-lined screw cap (Fisher; cat. 9998-15), 50 µg of internal standard was added, heptadecanoic acid (C17:0), followed by the serum sample. An aqueous phase (1.0 mL of 0.1 M KCl) was added, and total lipids were extracted by adding 4.0 mL chloroform: methanol (2:1, vol/vol), capping tightly, and vortexing for 1 min. Tubes were flushed with nitrogen gas and stored at 4 °C overnight to facilitate phase separation.

The following day, samples were vortexed and centrifuged at 1,460 rpm for 10 min at 21 °C to separate phases. Saponification was performed by adding 2.0 mL of 0.5 M KOH in methanol (prepared as 7.014 g KOH in 250 mL MeOH), vortexing briefly (5–10 s), and heating at 100 °C for 1 h in an oven or block, checking for evaporation every 15 min.

For methylation, 2.0 mL hexane and 2.0 mL of 14% boron-trifluoride in methanol were added, and tubes were heated at 100 °C for 1 h, again monitoring evaporation at 15 min intervals. After cooling for 10 min at room temperature, tubes were vortexed 1 min, and 1.0 mL double-distilled water was added to stop the reaction. Samples were centrifuged and the upper hexane layer was collected with a 5 ¾-in Pasteur pipette into clean 2 mL GC vials with PTFE caps.

For serum samples, methyl esters were reconstituted in 50 µL hexane for GC analysis (split injection) on an Agilent 7890/7890A GC as needed to avoid column overload; diet samples were reconstituted in 1.0 mL hexane and similarly diluted 1:5. Samples were either analyzed promptly on an SP-2560 fused-silica capillary column (100 m × 0.25 mm i.d. × 0.20 µm film).

### α-Tocopherol quantification

Whole blood was collected and spotted onto dried blood spot (DBS) cards, with 20 µL of blood applied per spot and two spots per card. After air drying at room temperature for a few minutes, the cards were stored at −20 °C in sealed containers until shipment. Samples were sent to Lipid Analytical Laboratories Inc. (Mississauga, Ontario, Canada) for α-Tocopherol analysis.

For extraction, one DBS spot per sample was punched and transferred to a 4 mL amber vial. A mixture of 100 µL water and 100 µL methanol was added, followed by 3 mL of methyl tert-butyl ether. The vial was vortexed briefly and then placed on a mechanical shaker for 30 minutes to extract fat-soluble components. After shaking, 1.5 mL of the organic layer was transferred to a second amber vial and evaporated under a stream of dry nitrogen gas. The residue was reconstituted in 300 µL of solution containing 150 µL internal standard solution (d_6_-α-tocopherol) and 150 µL methanol. A 250 µL aliquot of the supernatant was transferred to an amber high-performance liquid chromatography vial fitted with a conical glass insert for injection.

Calibration standards were prepared by spotting serial dilutions of α-tocopherol standard (Sigma-Aldrich, St. Louis, Missouri, United States of America). Calibration levels included 0, 1, 5, 25, 50, 100, 250, and 500 ppb. The calibration curve was validated for linearity, accuracy, and precision and was used to calculate final concentrations by comparing analyte and internal standard peak area ratios.

Chromatographic separation was performed using 120 EC-C18 column (2.1 × 100 mm, 2.7 µm particle size; Agilent InfinityLab Poroshell) maintained at 45 °C. The mobile phase consisted of water with 0.1% formic acid and 0.1 mM ammonium formate (Mobile Phase A) and methanol with the same additives (Mobile Phase B). The gradient began at 12% A and 88% B, progressed to 100% B by 7 min, and returned to initial conditions by 11 min. The flow rate was set to 0.6 mL/min and the injection volume was 8 µL.

Detection was carried out using an Agilent 6495 Triple Quadrupole LC-MS/MS system operated in Multiple Reaction Monitoring mode with Agilent Jet Stream electrospray ionization in positive mode. The monitored MRM transitions for α-tocopherol included a precursor ion with a mass-to-charge ratio (m/z) of 431.4, which was fragmented to produce two product ions: m/z 165.2, used as the quantifier, and m/z 137.2, used as the qualifier. For the internal standard, d_6_-α-tocopherol, the precursor ion was m/z 437.4, which yielded product ions of m/z 171.1 (quantifier) and m/z 143.1 (qualifier). The drying gas flow was set at 11 L/min at 250 °C, nebulizer pressure at 40 psi, sheath gas at 12 L/min at 350 °C, and capillary voltage at 4000 V. Nitrogen was used as the collision gas. Final concentrations of α-tocopherol were reported as µg/mL of whole blood equivalent.

Monounsaturated fatty acids (MUFA), saturated fatty acids (SFA), and PUFA were defined as the sum of their respective individual FA measured in serum lipid fractions. These values were manually summed from individual FA concentrations rather than directly measured as composite variables.

### Malondialdehyde concentration

Aliquots (200 µL) were thawed to allow duplicate MDA measurements, with 100 µL of serum used per replicate. MDA concentrations were quantified using a Canine MDA ELISA kit (MyBioSource Inc., San Diego, CA, USA) according to the manufacturer’s instructions. The assay has been previously validated for use in canine samples (Phungviwatnikul et al. 2020). The coefficient of variation (CV) between duplicate measurements was calculated as a measure of analytical precision. Duplicate measurements with CV values >20% were considered insufficiently precise for reliable quantification and were excluded from subsequent statistical analyses.

### Phospholipid separation by thin-layer chromatography

Serum aliquots (100 µL) were thawed and mixed with 1.0 mL of 0.1 M potassium chloride (KCl; Sigma-Aldrich, Oakville, Ontario, Canada) in a 15 mL acid-washed glass tube. The tube was then rinsed with a total of 4 mL of freshly prepared chloroform: methanol (2:1, v/v) in two 2 mL portions. After centrifugation, the lower chloroform phase was carefully collected using a glass Pasteur pipette and dried under a stream of nitrogen. Each dried lipid extract was then reconstituted in 100 µL of chloroform in preparation for thin-layer chromatography (TLC). High-performance TLC (HPTLC) plates (20 × 20 cm, H-grade) were activated by heating at 100 °C for 1 h, before sample application. The activated plates were scored into 2 cm-wide vertical lanes. Each 100 µL lipid sample was applied to a single lane using a 30 µL capillary tube with a rubber bulb. The TLC plates were placed vertically into the tank by resting the 1 cm edge in the solvent. The tank was covered with a lid and weighted to ensure a tight seal. The plates were developed until the solvent front reached approximately 1 cm from the top edge. In a fume hood or enclosed space with a cardboard box, the TLC plates were evenly sprayed with a fine mist of 0.1% (w/v) 1-amino-2-naphthol-4-sulfonic acid (ANSA) solution using a TLC nebulizer.

For methylation of the extracted lipids, 2 mL of freshly prepared 14% boron trifluoride-methanol (BF₃-MeOH; Sigma-Aldrich, Oakville, Ontario, Canada) was added. The samples were incubated at 100 °C for 90 min in a heat block. To stop methylation, 2 mL of double-distilled water was added, and the tubes were vortexed for 30 sec. The samples were then centrifuged at 270×g for 10 min at 21 °C to allow phase separation. The upper hexane layer was collected, dried under nitrogen, and reconstituted in 25 µL of hexane for gas chromatography (GC) analysis. Glass vials with GC inserts were used to avoid sample loss.

Analysis was performed using GC (Agilent 6890, Agilent Technologies, Mississauga, Ontario, Canada) equipped with a flame ionization detector.

The relative composition of FA was expressed as the percentage of the total peak area. Serum phospholipids are present predominantly as PC, LysoPC, and SM, and these classes were the most readily and consistently detected by thin-layer chromatography. Although phosphatidylethanolamine (PE) is also present in serum, its lower abundance and more limited detectability under these analytical conditions likely reduced consistent measurement; however, changes observed in PC are likely to be mirrored in PE composition.

### Cytokine profiling

To assess the inflammatory response to dietary EPA+DHA, we utilized a commercial multiplex canine cytokine assay (Milliplex Canine Cytokine Panel, EMD Millipore Corporation, Billerica, Massachusetts, United States of America), which includes 13 analytes. Serum aliquots (50 µL) were thawed to allow for duplicate measurements of cytokines, with 25 µL of serum used per replicate. The plate was run using a Bio-Plex 200 system with Bio-Plex Data pro Version 1.3 (Bio-Rad, Mississauga, Ontario, Canada). The quality control samples and standard curves provided in the kit were run according to the manufacturer’s protocol. The CV for each set of duplicates was evaluated, and if it was >20%, the results from that sample were discarded.

### Statistical analysis

Feed intake, BW, concentrations of phospholipid FA and FA fractions, α-tocopherol, MDA, and cytokines (IL-8, KC, and IL-18) were analyzed using PROC GLIMMIX in SAS (v. 9.4; SAS Institute Inc., Cary, North Carolina, United States of America). Dog was treated as a random effect, week as a repeated measure, and dose of EPA and DHA [DG: LOW (0.03 g/kg BW^0.75^), MOD (0.45 g/kg BW^0.75^), and HIGH (0.71 g/kg BW^0.75^)], week and their interactions were modelled as fixed effects. Pre-treatment baseline (wk 0) was used as a covariate for all the biomarkers except for MDA due to insufficient baseline sample availability. To assess the normality of residuals, the Shapiro–Wilk test was used, and we visually inspected the studentized residuals using Q-Q plots and histograms to ensure that the assumption of normality was met. Statistical significance was set at *P* ≤ 0.05, and trends when 0.05 < *P* ≤ 0.1. When fixed effects were significant, mean separation was performed using the Tukey–Kramer method.

## Results

### Body weight and feed intake

Week effects were observed for FI (g/day) (*P* < 0.01), with FI lower at wk 2 (244.0 g/day), unchanged between weeks 2 and 3, and then gradually increased from week 4 (261.8 g/day) through week 8 (455.3 g/day). In contrast, BW remained relatively stable throughout the study period (20.8 kg to 21.5 kg, *P* = 0.286). There were no differences among DG in BW (*P* = 0.369) and FI (*P* = 0.123) (Table S2).

### Lipid fractions: phosphatidylcholine

In the PC fraction, all FA are reported in Table S3 and summarized in Table 1. Pooling weeks 4 and 8, EPA (20:5n-3), DHA (22:6n-3), total n-3 FA, and EPA+DHA (20:5n-3 + 22:6n-3) were lowest in LOW, whereas MOD and HIGH did not differ (*P* < 0.05 for all). Docosapentaenoic acid (DPA, 22:5n-3) was greater in LOW (1.80% total FA) than in MOD (2.20% total FA) and HIGH (2.23% total FA), with MOD and HIGH being similar (*P* = 0.03). Although total PUFA did not differ, the n-6: n-3 ratio and AA: EPA+DHA ratio were greatest in LOW (5.60:1), with MOD (1.63:1) and HIGH (1.84:1) being similar (*P* < 0.05 for both).

**Table 1:** Least squares means (LSMeans ± SEM) for serum fatty acids (%) in phosphatidylcholine (PC) across weeks (0, 4, and 8) and dose groups (DG) of eicosapentaenoic acid (EPA) and docosahexaenoic acid (DHA) [LOW (0.03 g/kg BW^0.75^), MOD (0.45 g/kg BW^0.75^), and HIGH (0.71 g/kg BW^0.75^)] over 8 weeks, with corresponding *P*-values for main (DG, week) and interaction (DG × week) effects. Week 0 (pre-treatment baseline) was used as a covariate, and Mean values represent LSMeans averaged over weeks 4 and 8 (baseline-adjusted). Different superscript letters (^a, b^) indicate statistically significant differences among DGs (*P* ≤ 0.05), and different subscript letters (_a, b_) indicate statistically significant differences in DG × Week interaction (*P* ≤ 0.05).

Fatty acid in PC (%)		Week				*P*-value		
		0	4	8	Mean	DG	Week	DG × Week
**20:5n3 (EPA)**	LOW	0.90 ± 0.05	1.45 ± 0.90	2.77 ± 0.9	2.11 ± 0.76^b^	<0.01	0.96	0.23
	MOD	0.81 ± 0.05	7.26 ± 0.90	7.19 ± 0.90	7.22 ± 0.75^a^			
	HIGH	0.84 ± 0.05	8.67 ± 0.89	7.50 ± 0.89	8.08 ± 0.74^a^			
**22:6n3 (DHA)**	LOW	2.78 ± 0.14	2.44 ± 0.32	2.15 ± 0.32	2.29 ± 0.27^b^	<0.01	<0.01	0.10
	MOD	2.46 ± 0.14	4.43 ± 0.32	3.94 ± 0.32	4.19 ± 0.27^a^			
	HIGH	2.65 ± 0.14	4.83 ± 0.31	3.57 ± 0.31	4.20 ± 0.27^a^			
**18:3n3 (ALA)**	LOW	0.31 ± 0.05	0.29 ± 0.02	0.32 ± 0.02	0.30 ± 0.01	0.17	0.02	0.45
	MOD	0.31 ± 0.05	0.24 ± 0.02	0.30 ± 0.02	0.27 ± 0.01			
	HIGH	0.27 ± 0.05	0.26 ± 0.02	0.28 ± 0.02	0.27 ± 0.01			
**20:4n6 (AA)**	LOW	17.85 ± 0.49	19.13 ± 0.73	18.05 ± 0.73	18.59 ± 0.59^a^	<0.01	0.30	0.28
	MOD	18.28 ± 0.49	16.05 ± 0.73	14.89 ± 0.73	15.47 ± 0.59^b^			
	HIGH	18.11 ± 0.49	14.16 ± 0.73	14.79 ± 0.73	14.47 ± 0.59^b^			
**n-6: n-3**	LOW	7.02 ± 0.31	7.16 ± 0.74	7.87 ± 0.74	7.51 ± 0.58^a^	<0.01	0.14	0.49
	MOD	7.62 ± 0.31	2.48 ± 0.72	2.56 ± 0.72	2.52 ± 0.57^b^			
	HIGH	7.71 ± 0.31	2.13 ± 0.72	3.74 ± 0.72	2.94 ± 0.57^b^			
**AA: EPA+DHA**	LOW	4.96 ± 0.45	5.13 ± 0.66	6.07 ± 0.66	5.60 ± 0.51^a^	<0.01	0.10	0.44
	MOD	5.14 ± 0.45	1.62 ± 0.66	1.63 ± 0.66	1.63 ± 0.51^b^			
	HIGH	5.24 ± 0.45	1.06 ± 0.65	2.62 ± 0.65	1.84 ± 0.49^b^			

Among n-6 FA, linoleic acid (18:2n6), arachidonic acid (20:4n6), adrenic acid (22:4n6), docosapentaenoic acid (22:5n6), and total n-6 FA were greatest in LOW, whereas MOD and HIGH did not differ (*P* < 0.05 for all). A DG × week interaction was also detected for dihomo-γ-linolenic acid (20:3n-6; *P* < 0.01), which reflected a reduction over time in HIGH relative to baseline.

Small changes were also observed in other FA, shown in Table S3. DG × week interactions were detected for stearic acid (18:0) and nonadecanoic acid (19:0) (*P* < 0.05 for both), although no between-group differences were detected at individual time points for stearic acid (18:0; *P* = 0.54) or nonadecanoic acid (19:0; *P* = 0.49). In addition, arachidic acid (20:0; *P* = 0.02) was greatest in HIGH (0.13 % total FA), which was greater than LOW (0.10 % total FA) and similar to MOD (0.11 % total FA), with LOW and MOD being similar. Lignoceric acid (24:0) and erucic acid (22:1n9) were greatest in HIGH, which was greater than MOD and similar to LOW, and LOW and MOD were similar (*P* < 0.05 for both).

### Lipid fractions: lysophosphatidylcholine

In the LysoPC fraction, all FA are reported in Table S4 and summarized in Table 2. No DG × week interaction was detected for any measured FA or for the n-6: n-3 ratio (*P* > 0.05 for all). For the overall effect of DG, pooled across timepoints, ALA (18:3n-3), EPA (20:5n-3), EPA+DHA, and total n-3 FA were lowest in MOD, which was lower than HIGH (all, *P* < 0.05), with LOW and HIGH not differing. Although total PUFA did not differ (*P* = 0.18), the n-6: n-3 ratio and AA: EPA+DHA ratio were greatest in LOW, with MOD and HIGH being similar (*P* < 0.05 for both).

**Table 2:** Least squares means (LSMeans ± SEM) for serum fatty acids (%) in lysophosphatidylcholine (lysoPC) of dogs (n = 27) across weeks (0, 4, and 8) and dose groups (DG) of eicosapentaenoic acid (EPA) and docosahexaenoic acid (DHA) [LOW (0.03 g/kg BW^0.75^), MOD (0.45 g/kg BW^0.75^), and HIGH (0.71 g/kg BW^0.75^)] over 8 wk, with corresponding *P*-values for main (DG, week) and interaction (DG × week) effects. Week 0 (pre-treatment baseline) was used as a covariate, and Mean values represent LSMeans averaged over weeks 4 and 8 (baseline-adjusted). Different superscript letters (^a, b^) indicate statistically significant differences among DGs (*P* ≤ 0.05).

Fatty acid in LysoPC (%)		Week				*P*-value		
		0	4	8	Mean	DG	Week	DG × Week
**20:5n3 (EPA)**	LOW	0.19 ± 0.09	0.93 ± 0.20	0.21 ± 0.20	0.57 ± 0.16^b^	<0.01	0.04	0.14
	MOD	0.03 ± 0.09	1.91 ± 0.20	1.73 ± 0.20	1.82 ± 0.16^a^			
	HIGH	0.00 ± 0.09	2.11 ± 0.20	2.07 ± 0.20	2.09 ± 0.16^a^			
**22:6n3 (DHA)**	LOW	1.03 ± 0.30	1.04 ± 0.22	0.84 ± 0.22	0.94 ± 0.16	0.23	0.97	0.62
	MOD	0.49 ± 0.30	1.07 ± 0.22	1.29 ± 0.22	1.18 ± 0.16			
	HIGH	0.84 ± 0.30	1.35 ± 0.22	1.32 ± 0.22	1.33 ± 0.16			
**18:3n3 (ALA)**	LOW	0.00 ± 0.00	0.22 ± 0.10	0.00 ± 0.10	0.11 ± 0.07^ab^	0.05	<0.01	0.05
	MOD	0.00 ± 0.00	0.09 ± 0.10	0.00 ± 0.10	0.04 ± 0.07^b^			
	HIGH	0.00 ± 0.00	0.60 ± 0.10	0.00 ± 0.10	0.30 ± 0.07^a^			
**20:4n6 (AA)**	LOW	4.23 ± 0.33	4.71 ± 0.33	4.50 ± 0.33	4.61 ± 0.22^a^	<0.01	0.26	0.96
	MOD	4.08 ± 0.33	3.78 ± 0.33	3.37 ± 0.33	3.57 ± 0.22^b^			
	HIGH	4.13 ± 0.33	3.42 ± 0.33	3.06 ± 0.33	3.24 ± 0.22^b^			
**n-6: n-3**	LOW	4.92 ± 2.03	6.06 ± 1.00	9.66 ± 1.19	7.86 ± 0.71^a^	<0.01	0.08	0.52
	MOD	4.20 ± 2.03	2.93 ± 1.20	4.35 ± 1.20	3.64 ± 0.73^b^			
	HIGH	6.66 ± 2.03	3.01 ± 1.09	3.93 ± 1.09	3.47 ± 0.69^b^			
**AA: EPA+DHA**	LOW	2.43 ± 0.87	2.76 ± 0.49	4.25 ± 0.59	3.51 ± 0.34^a^	<0.01	<0.01	<0.01
	MOD	2.17 ± 0.87	1.37 ± 0.61	1.36 ± 0.61	1.37 ± 0.37^b^			
	HIGH	2.09 ± 0.87	1.02 ± 0.54	1.03 ± 0.54	1.02 ± 0.32^b^			

Among n-6 FA, LA (18:2n6), dihomo-gamma-linolenic acid (20:3n6), arachidonic acid (20:4n6), and total n-6 FA were greatest in LOW, which was greater than HIGH and similar to MOD, with MOD and HIGH being similar (*P* < 0.05 for all).

Small changes were also observed over the week. Pooled across treatments, palmitic acid (16:0), nonadecanoic acid (19:0), arachidic acid (20:0), tricosanoic acid (23:0), lignoceric acid (24:0), gondoic acid (20:1n-9), DGLA (20:3n-6), ALA (18:3n-3), and EPA (20:5n-3) were greater at week 4 than week 8 (*P* < 0.05 for all), whereas oleic acid (18:1n-9), LA (18:2n6), mead acid (20:3n9), and the n-6: n-3 ratio were greater at week 8 than week 4 (*P* < 0.05 for all).

### Lipid fractions: sphingomyelin

In the SM fraction, all FA are reported in Table S5 and summarized in Table 3. No DG × week interaction was detected for any measured FA (*P* > 0.05 for all). For the overall effect of DG, pooled across weeks, EPA (20:5n-3) and EPA+DHA were greatest in HIGH, which was greater than LOW and similar to MOD, whereas MOD was similar to LOW (*P* < 0.05 for both). The n-6: n-3 ratio and AA: EPA+DHA ratio were greatest in LOW, which was greater than HIGH and similar to MOD, whereas MOD did not differ from HIGH (*P* < 0.05 for both).

**Table 3:** Least squares means (LSMeans ± SEM) for serum fatty acids (%) in sphingomyelin (SM) of dogs (n = 27) across weeks (0, 4, and 8) and dose groups (DG) of eicosapentaenoic acid (EPA) and docosahexaenoic acid (DHA) [LOW (0.03 g/kg BW^0.75^), MOD (0.45 g/kg BW^0.75^), and HIGH (0.71 g/kg BW^0.75^)] over 8 wk, with corresponding *P*-values for main (DG, week) and interaction (DG × week) effects. Week 0 (pre-treatment baseline) was used as a covariate, and Mean values represent LSMeans averaged over weeks 4 and 8 (baseline-adjusted). Different superscript letters (^a, b^) indicate statistically significant differences among DGs (*P* ≤ 0.05).

Fatty acid in SM (%)		Week				*P*-value		
		0	4	8	Mean	DG	Week	DG × Week
**20:5n3 (EPA)**	LOW	0.05 ± 0.06	1.31 ± 0.36	0.31 ± 0.36	0.81 ± 0.27^b^	<0.01	<0.01	0.42
	MOD	0.00 ± 0.06	1.69 ± 0.36	1.30 ± 0.36	1.50 ± 0.28^a,b^			
	HIGH	0.15 ± 0.06	2.80 ± 0.37	1.56 ± 0.37	2.18 ± 0.28^a^			
**22:6n3 (DHA)**	LOW	0.31 ± 0.16	1.13 ± 0.23	0.46 ± 0.23	0.80 ± 0.18	0.53	<0.01	0.66
	MOD	0.18 ± 0.16	1.23 ± 0.23	0.59 ± 0.23	0.91 ± 0.18			
	HIGH	0.46 ± 0.16	1.57 ± 0.23	0.60 ± 0.23	1.09 ± 0.18			
**20:4n6 (AA)**	LOW	2.45 ± 0.53	6.64 ± 1.09	4.08 ± 1.09	5.36 ± 0.86	0.24	<0.01	0.99
	MOD	2.45 ± 0.53	4.98 ± 1.09	2.68 ± 1.09	3.83 ± 0.86			
	HIGH	3.42 ± 0.53	4.52 ± 1.11	2.03 ± 1.11	3.27 ± 0.86			
**18:3n3 (ALA)**	LOW	0.08 ± 0.10	0.35 ± 0.09	0.24 ± 0.09	0.29 ± 0.05	0.17	0.17	0.99
	MOD	0.24 ± 0.10	0.23 ± 0.09	0.13 ± 0.09	0.18 ± 0.06			
	HIGH	0.04 ± 0.10	0.22 ± 0.09	0.09 ± 0.09	0.16 ± 0.05			
**n-6: n-3**	LOW	3.32 ± 2.05	6.92 ± 1.24	11.02 ± 1.32	9.46 ± 0.86^a^	0.01	0.08	0.18
	MOD	5.66 ± 2.05	4.63 ± 1.17	5.43 ± 1.17	7.60 ± 0.75^a,b^			
	HIGH	3.56 ± 2.05	3.91 ± 1.17	4.71 ± 1.32	5.57 ± 0.78^b^			
**AA: EPA+DHA**	LOW	1.21 ± 0.61	3.24 ± 0.48	4.16 ± 0.68	3.56 ± 0.41^a^	0.05	0.63	0.20
	MOD	0.84 ± 0.61	1.68 ± 0.46	1.34 ± 0.46	2.28 ± 0.42^a,b^			
	HIGH	1.27 ± 0.61	1.03 ± 0.46	1.01 ± 0.51	1.61 ± 0.37^b^			

Changes in n-6 FA were also observed by week. Pooled across treatments, LA (18:2n6), DGLA (20:3n6), AA (20:4n6), and total n-6 FA were greater at week 4 than week 8 (*P* < 0.05 for all).

Additional changes were observed in other FA over week. Stearic acid (18:0), palmitoleic acid (16:1n7), vaccenic acid (18:1n7), oleic acid (18:1n9), EPA (20:5n-3), docosapentaenoic acid (22:5n-3), DHA (22:6n-3), total n-3 FA, EPA+DHA, and total PUFA were greater at week 4 than week 8 (*P* < 0.05 for all). In contrast, arachidic acid (20:0), behenic acid (22:0), tricosanoic acid (23:0), lignoceric acid (24:0), total SFA, erucic acid (22:1n9), nervonic acid (24:1n9), and total MUFA were greater at week 8 than week 4 (*P* < 0.05 for all).

### Total FA incorporation

All values for total serum FA are reported in Table S6 and summarized in Table 4. No DG × week interaction effects were detected for any of the measured serum total FA (*P* > 0.05 for all; Table S6). For the overall effect of dietary treatment, pooled across weeks 4 and 8, concentrations of EPA (20:5n-3), DHA (22:6n-3), total n-3 PUFA, and EPA+DHA were lowest in the LOW group and greater in MOD and HIGH, which did not differ (*P* < 0.05 for all). In contrast, LA (18:2n-6), DGLA (20:3n-6), AA (20:4n-6), adrenic acid (22:4n-6), and total n-6 PUFA were greatest in LOW and lower in MOD and HIGH, which were not different from each other (*P* < 0.05 for all). Consequently, the n-6: n-3 and AA: EPA+DHA ratios were greatest in LOW and lower in MOD and HIGH, which did not differ (*P* < 0.05 for both).

**Table 4:** Least squares means (LSMeans ± SEM) for total fatty acid (FA) content (ul/mL serum) of dogs (*n* = 27) across weeks (0, 4, and 8) and dose groups (DG) of eicosapentaenoic acid (EPA) and docosahexaenoic acid (DHA) [LOW (0.03 g/kg BW^0.75^), MOD (0.45 g/kg BW^0.75^), and HIGH (0.71 g/kg BW^0.75^)] over 8 wk, with corresponding *P*-values for main (DG, week) and interaction (DG × week) effects. Week 0 (pre-treatment baseline) was used as a covariate, and Mean values represent LSMeans averaged over weeks 4 and 8 (baseline-adjusted). Different superscript letters (^a, b^) indicate statistically significant differences among DGs (*P* ≤ 0.05).

Total FA (ul/mL serum)		Week				*P*-value		
		0	4	8	Mean	DG	Week	DG × Week
**20:5n3 (EPA)**	LOW	5.87 ± 0.94	8.03 ± 3.64	6.15 ± 0.51	6.63 ± 1.14^b^	<0.01	0.01	0.12
	MOD	5.15 ± 0.76	19.43 ± 3.67	47.84 ± 4.85	24.33 ± 3.81^a,b^			
	HIGH	5.44 ± 0.57	45.03 ± 11.72	67.63 ± 10.72	67.63 ± 10.72^a^			
**22:6n3 (DHA)**	LOW	13.43 ± 1.74	10.34 ± 3.31	11.13 ± 1.23	11.68 ± 1.22^b^	<0.01	0.01	0.14
	MOD	11.25 ± 1.77	9.81 ± 2.44	22.35 ± 2.04	14.88 ± 1.33^b^			
	HIGH	11.96 ± 1.03	20.54 ± 4.89	25.77 ± 2.85	19.42 ± 2.15^a^			
**20:4n6 (AA)**	LOW	102.59 ± 11.32	121.72 ± 30.77	144.82 ± 15.37	114.17 ± 11.81^a^	0.02	<0.01	0.28
	MOD	97.08 ± 16.76	37.30 ± 7.10	88.12 ± 8.78	77.76 ± 7.74^b^			
	HIGH	99.66 ± 8.42	99.66 ± 8.42	83.28 ± 7.20	86.63 ± 8.71^b^			
**18:3n3 (ALA)**	LOW	4.14 ± 0.37	3.59 ± 1.13	6.95 ± 0.99	4.94 ± 0.56^a^	0.06	<0.01	0.42
	MOD	3.54 ± 0.59	1.66 ± 0.30	5.25 ± 0.71	3.61 ± 0.40^b^			
	HIGH	3.56 ± 0.24	3.21 ± 0.84	4.89 ± 0.37	3.89 ± 0.34^b^			
**n-6: n-3**	LOW	10.24 ± 0.44	10.28 ± 0.65	12.91 ± 0.64	11.08 ± 0.41^a^	<0.01	<0.63	0.34
	MOD	9.43 ± 1.17	3.15 ± 0.63	3.27 ± 0.25	5.66 ± 0.69^b^			
	HIGH	11.02 ± 0.27	3.06 ± 0.19	2.70 ± 0.19	5.60 ± 0.76^b^			
**AA: EPA+DHA**	LOW	5.45 ± 0.33	5.58 ± 0.54	8.38 ± 0.42	6.44 ± 0.37^a^	<0.01	<0.01	0.27
	MOD	5.26 ± 0.66	1.27 ± 0.13	1.28 ± 0.13	2.81 ± 0.43^b^			
	HIGH	5.80 ± 0.23	1.12 ± 0.07	0.95 ± 0.07	2.61 ± 0.45^b^			

For the overall effect by week, pooled across dietary treatments, EPA (20:5n-3), DHA (22:6n-3), total n-3 PUFA, and EPA+DHA were greater at week 4 than week 8 (*P* < 0.05 for all). In contrast, AA (20:4n-6), total n-6 PUFA, and total MUFA were greater at week 8 than at week 4 (*P* < 0.05 for all). The AA: DHA+EPA ratio differed among dietary treatments (*P* < 0.01), being greatest in LOW and lower in MOD and HIGH, which did not differ from each other.

### α-Tocopherol concentrations

**Figure 1:**
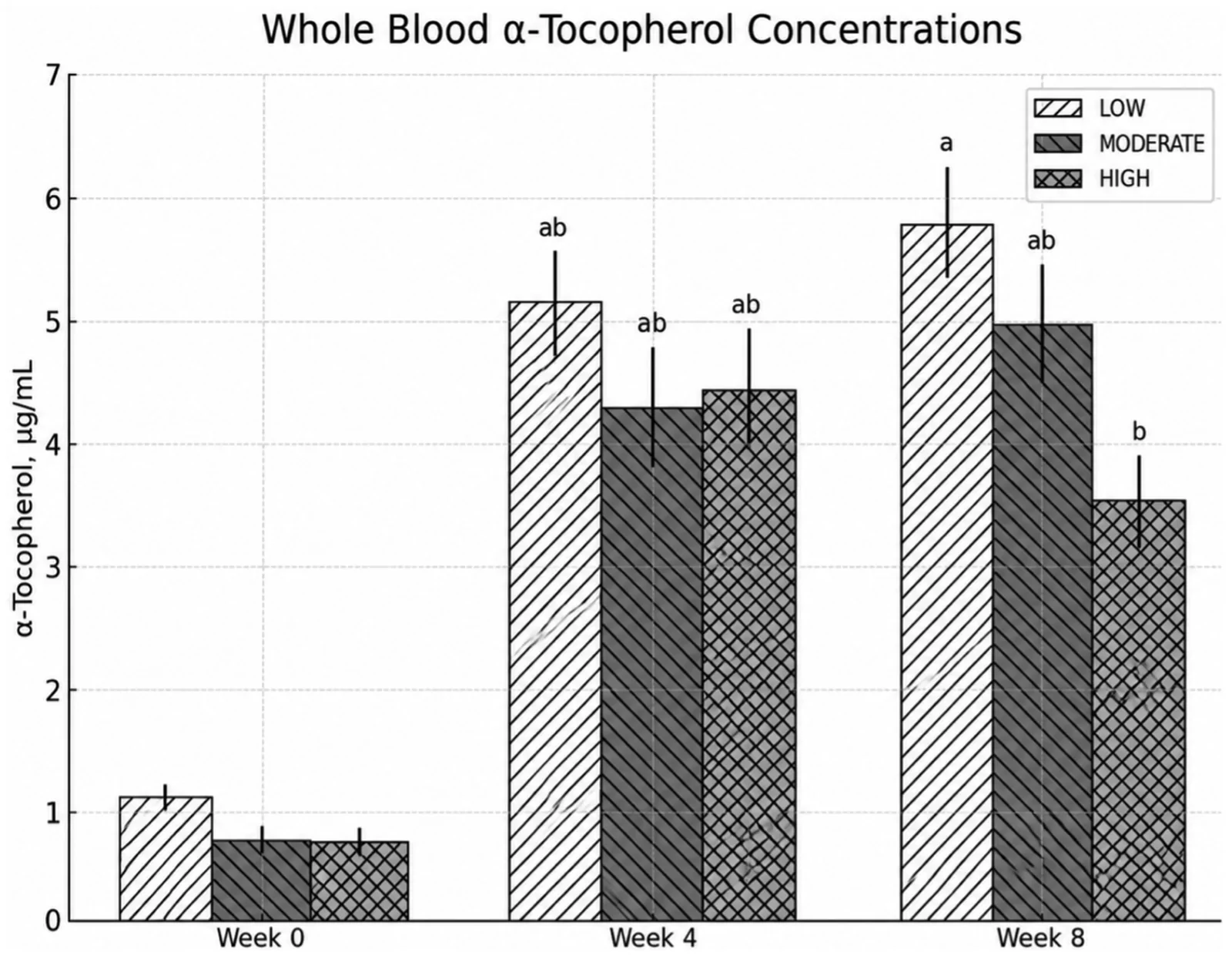
Least squares means (LSMeans ± SEM) for whole blood α-tocopherol concentrations (µg/mL) of dogs (*n* = 27) across weeks (0, 4, and 8) and experimental dose group (DG) of(eicosapentaenoic acid (EPA) and docosahexaenoic acid (DHA)) [LOW (0.03 g/kg BW^0.75^), MOD (0.45 g/kg BW^0.75^), and HIGH (0.71 g/kg BW^0.75^)] over 8 wk. Week 0 (pre-treatment baseline) was used as a covariate. Different superscripts (^a, b^) indicate statistically significant differences (*P* ≦ 0.05).

A DG × week interaction was observed for whole blood α-tocopherol concentration in week 8 between LOW and HIGH (Figure 1) (*P* = 0.034). At week 8, α-tocopherol concentrations were greatest in LOW (5.82 µg/mL), which was higher than in HIGH (3.52 µg/mL) and similar to MOD (5.00 µg/mL); MOD and HIGH did not differ significantly from each other. There were no differences among treatments at week 4.

### Malondialdehyde concentrations

There was no effect of the DG × week interaction (*P* = 0.53) or DG (*P* = 0.35) on MDA concentrations (Table 5). There was no effect of week, but there was a trend toward an increase by week from week 4 (1.8 mmol/mL) to week 8 (2.1 mmol/mL) (*P* = 0.05).

**Table 5:** Least squares means (LSMeans ± SEM) for serum malondialdehyde (MDA) concentrations (mmol/mL) of dogs (*n* = 27) across weeks (4 and 8) and experimental dose groups (DG) of eicosapentaenoic acid (EPA) and docosahexaenoic acid (DHA) [(LOW (0.03 g/kg BW^0.75^), MOD (0.45 g/kgBW^0.75^), and HIGH (0.71 g/kg BW^0.75^)] over 8 wk, with corresponding *P*-values for main (DG, week) and interaction (DG × week) effects.

	DG			Week		*P*-value		
	LOW	MOD	HIGH	4	8	DG	Week	DG × Week
**MDA**	2.0 ± 0.1	2.0 ± 0.1	1.8 ± 0.1	1.8 ± 0.1	2.1 ± 0.1	0.35	0.05	0.54

### Cytokine concentrations

Among the 13 cytokines measured, only IL-8, KC-like, and IL-18 were consistently detectable. The remaining analytes failed to meet the optical density ratio thresholds, indicating that their concentrations were below the lowest standard detection limits and therefore not quantifiable. An effect of the week was observed for concentrations of KC-like and IL-18 cytokines (*P* < 0.05, Table 6). Concentrations of both were greater at week 8 (KC-Like = 124.0 mmol/mL, IL-18 = 48.2 mmol/mL) than at week 4 (KC-Like = 100.4 mmol/mL, IL-18 = 36.7 mmol/mL) when pooled among treatments. There were no effects of DG or DG × week for KC-like or IL-18 concentrations, and no impact of DG, time, or interaction on IL-8 (*P* > 0.05 for all).

**Table 6:** Least squares means (LSMeans ± SEM) for serum cytokines [Interleukin-8 (IL-8), Keratinocyte-derived chemokine (KC-like), and Interleukin-18 (IL-18)] concentrations (mmol/mL) of dogs (*n* = 27) across weeks (0, 4, and 8) and experimental dose groups (DG) of eicosapentaenoic acid (EPA) and docosahexaenoic acid (DHA) [(LOW (0.03 g/kg BW^0.75^), MOD (0.45 g/kg BW^0.75^), or HIGH (0.71 g/kg BW^0.75^)] over 8 wk, with corresponding *P*-values for main (DG, week) and interaction (DG × week) effects. Week 0 was used as a covariate. Different superscripts (^a, b^) indicate statistically significant differences (*P* ≦ 0.05).

	DG			Week			*P*-value		
	LOW	MOD	HIGH	0	4	8	DG	Week	DG × Week
**IL-8**	4296.7 ± 277.4	4328.5 ± 272.1	3873.1 ± 272.0	4897.2 ± 304.2	4268.0 ± 178.0	4064.1 ± 178.0	0.43	0.25	0.51
**KC-like**	118.9 ± 16.5	105.8 ± 16.3	111.9 ± 16.3	128.9 ± 86.2	100.4 ± 10.3^b^	124.0 ± 10.5^a^	0.85	0.01	0.13
**IL-18**	47.5 ± 3.4	39.2 ± 3.6	40.5 ± 3.6	36.0 ± 13.4	36.7 ± 2.2^b^	48.2 ± 2.3^a^	0.23	<0.01	0.89

## Discussion

Based on unchanged BW and FI among dose groups and no increase in MDA or inflammatory cytokines, feeding EPA and DHA at 0.71 g/kg BW^0.75^ was well tolerated in healthy adult Siberian and Alaskan husky dogs. These findings support further work to determine whether higher EPA and DHA intake translates into functional benefits, particularly in outcomes such as cognitive function and neuromuscular performance, rather than changes in circulating FA status alone. This higher dose resulted in measurable incorporation of EPA and DHA into phospholipids without adverse oxidative or inflammatory effects, suggesting that the existing SUL may be overly conservative, similar to recent evidence in humans (Lust et al. 2023). Given that no previous canine studies have quantified EPA and DHA across discrete serum lipid fractions before Templeman et al. (2021), this linear response in SM provides important new insight into how structural membrane lipids may respond differently to higher long-chain n-3 PUFA provision. The capacity to safely reach higher tissue DHA incorporation in dogs mirrors observations in humans, where elevated DHA intake enhanced the n-3 index at doses that were previously thought to reach steady state (Lust et al. 2023). When viewed across all measured outcomes in this study, including lipid fractions, antioxidant status, inflammatory cytokines, and oxidative markers, the collective response indicates that highly active adult dogs can safely accommodate higher long-chain n-3 intake.

Total FA profiles demonstrated an increase in EPA and DHA, accompanied by lower total n-6 PUFA and a reduced AA: DHA+EPA ratio. These results agree with previous canine work where dietary manipulation of the n-6: n-3 PUFA ratio altered circulating lipid composition, eicosanoid balance, and oxidative status (Wander et al. 1997; Hall et al. 2003; Burron et al. 2023). Similarly, Templeman et al. (2021) reported that dietary FA composition strongly influences serum lipid fractions in sled dogs over 12 wk of feeding, noting that total FA measures change more gradually than specific phospholipid pools, which are more responsive to diet and exercise. Similar increases in DHA and total n-3 PUFA, alongside reductions in the n-6: n-3 ratio, have been observed in humans, livestock, and companion animals over similar durations of supplementation (Thanh et al. 2015; Lust et al. 2023; Burron et al. 2024).

Overall, the present findings suggest that most lipid pools reached a metabolic steady state by week 4 of supplementation, with little additional enrichment thereafter despite continued EPA and DHA intake. This pattern is consistent with previous canine studies demonstrating that incorporation of dietary long-chain n-3 PUFA into circulating lipid pools largely plateaus after wk 4 of fish oil supplementation (Hall et al. 2003).

Lipid fractions, however, differ in turnover, which influences both where EPA and DHA are incorporated and how quickly their physiological effects may be reflected (Bent and Bell 1995). As reviewed by Fajardo et al. (2011), PC is a dynamic membrane phospholipid pool that readily incorporates dietary FA and typically reflects shorter-term changes in intake over approximately 3 to 21 d, suggesting that this fraction becomes enriched rapidly because of its high turnover. The concurrent increase in EPA+DHA and reduction in AA and total n-6 PUFA within PC are consistent with a shift toward anti-inflammatory lipid synthesis (Wander et al. 1997).

Similar to PC, LysoPC appears to represent a relatively dynamic glycerophospholipid pool that integrates dietary long-chain n-3 PUFA readily and reflects more immediate changes in circulating and peripheral membrane reservoirs. In our dogs, the observed enrichment of EPA and DHA in LysoPC, together with the response seen in PC, suggests that these glycerophospholipid fractions are saturated more quickly and at lower doses than SM, likely because of their faster turnover and greater metabolic accessibility, and short-term adjustments in membrane-fraction incorporation (Bent and Bell 1995; Pasternak and Friedrichs 1970; Fajardo et al. 2011).

In contrast, SM is a more structurally rigid membrane lipid with slower turnover, providing insight into longer-term or more sustained FA integration into cell membranes (Fajardo et al. 2011). Mechanistic studies demonstrate that SM turns over more slowly than PC, consistent with a longer-lived structural membrane pool (Pasternak and Friedrichs 1970; Jungalwala 1974). Although the functional effects of EPA and DHA incorporation into SM fractions remain unclear in dogs, SM is known to play an important role in membrane organization and cell signaling (Chakraborty and Jiang 2013; Zhukov and Vereshchagin 2024). SM hydrolysis facilitates Toll-like receptor 4 clustering and enhances immune signaling, while lower ceramide concentration in fat-1 mice with higher endogenous n-3 PUFA suggests that n-3 incorporation into SM-rich membrane regions may help dampen inflammatory signaling (Chakraborty and Jiang 2013; Mondal et al. 2021; Zhukov and Vereshchagin 2024). However, the present results indicate fraction-specific long-chain n-3 PUFA incorporation rather than demonstrable systemic anti-inflammatory effects.

Because membrane remodeling influences oxidative balance and antioxidant demand, it is also important to consider how α-tocopherol status responds to increasing EPA and DHA intake. We observed a clear increase in whole blood α-tocopherol from baseline to week 4 across all treatment groups, which was expected given that the experimental diet (1888.65 IU/kg) contained more than double the α-tocopherol of the acclimation diet (775 IU/kg). Although all dogs across DG consumed the same dietary α-tocopherol concentration, whole blood α-tocopherol concentrations were lower by week 8 in the HIGH group, indicating greater utilization and suggesting that higher EPA and DHA intake may increase antioxidant demand. However, the present study also measured α-tocopherol in whole blood rather than serum, precluding direct comparison with published serum reference values. Hall et al. (2003) and Wander et al. (1997) both observed reduced circulating α-tocopherol in dogs fed higher fish-oil diets above the SUL for DHA+EPA, indicating that higher long-chain n-3 PUFA exposure increases reliance on antioxidant defenses. Notably, those studies reported this effect at lower fish-oil doses than the dosage point at which it became evident in the present study; however, the current study incorporated greater dietary α-tocopherol. The higher α-tocopherol concentration compared to other commercial diets likely provided sufficient antioxidant capacity at moderate EPA+DHA intake, delaying the decline until the greatest supplementation. Together, these results reinforce the principle that, as dietary PUFA intake increases, so does the requirement for antioxidant protection. Because EPA and DHA contain multiple double bonds, they are particularly susceptible to lipid peroxidation. As the principal lipid-soluble chain-breaking antioxidant, α-tocopherol scavenges lipid peroxyl radicals and terminates oxidative chain reactions, thereby protecting membrane lipids from oxidative damage. Consequently, greater incorporation of EPA and DHA into circulating lipids may increase α-tocopherol utilization despite stable MDA concentrations, suggesting that antioxidant defenses were sufficient to maintain oxidative balance. Ensuring adequate α-tocopherol relative to EPA+DHA intake is therefore critical, although additional dietary antioxidants may also help preserve antioxidant capacity and warrant further investigation (Hall et al. 2006).

Taken together, this suggests that greater EPA and DHA incorporation was not associated with detectable increases in lipid peroxidation. However, dietary α-tocopherol content reflected only a single time point and likely declined over storage. As such, lower whole blood α-tocopherol may have reflected reduced dietary exposure over time, rather than EPA and DHA dosage alone. Similar outcomes have been reported in dogs supplemented with fish oil, where α-tocopherol decreased without corresponding increases in oxidative markers (LeBlanc et al. 2005), and in working police dogs fed EPA- and DHA-rich diets (EPA ≈ 2.7 vs 0.04; DHA ≈ 0.5 vs 0.13) compared with dogs fed low n-3 diets, consistent with the present study (Ravic et al. 2022). In contrast, rabbits supplemented with fish oil showed both greater MDA and lower α-tocopherol (Mattioli et al. 2021), illustrating that oxidative stress becomes detectable when antioxidant supply does not keep pace with PUFA-driven demand or when oxidative stress in itself is more prevalent. Taken together, increased antioxidant utilization, rather than overt lipid peroxidation, characterized the response to higher EPA and DHA intake, and adequate dietary α-tocopherol can effectively buffer moderate oxidative stress.

Having established that oxidative stress did not increase despite greater antioxidant utilization, subsequent examination was conducted to determine whether this pattern extended to markers of systemic inflammation, where only three of the 13 cytokines analyzed were detectable. These results were consistent with previous findings in healthy dogs due to the low baseline immune activation and limited sensitivity of multiplex assays (Richter et al. 2018). The detected analytes, IL-8, KC-like, and IL-18, are associated with neutrophil recruitment and Th1-type inflammatory signaling (Argyl et al. 1999; Karlsson et al. 2016; Galan et al. 2018). While IL-8 remained stable, week-dependent increases in KC-like and IL-18 occurred independently of dietary EPA+DHA, likely reflecting normal physiological rhythms rather than treatment effects. Seasonal changes in daylight and temperature, or shifts in feed intake and metabolism, may have influenced immune signaling via neuroendocrine and metabolic pathways (Kaushik and Kaur 2003; Dopico et al. 2015). Given that no immune challenge was introduced, the lack of dietary effects on cytokine concentrations was expected and aligns with other studies in healthy dogs, where measurable immune responses typically occur only under inflammatory or disease conditions (Bauer 2011; Verbrugghe et al. 2014; Souza et al. 2018).

The present study has several limitations. All dogs were healthy adult Siberian Huskies housed under the same ownership and management, which minimized environmental variability and strengthened internal validity but may limit the generalizability of the findings to other breeds, life stages, or management conditions. Additionally, the absence of an inflammatory or physiological challenge may have limited our ability to detect the immunomodulatory effects of dietary EPA and DHA on circulating cytokine concentrations. The 8-wk study duration evaluated only short-term responses, and oxidative status was assessed using selected biomarkers rather than a comprehensive panel of oxidative and antioxidant indices. Furthermore, α-tocopherol was measured in whole blood rather than serum, precluding direct comparison with published serum reference values, and the relatively high vitamin E concentration of the experimental diet may have attenuated oxidative responses to increasing EPA and DHA intake. Future studies should evaluate longer supplementation periods across more diverse canine populations while incorporating inflammatory challenge models, additional mechanistic biomarkers, and tissue FA analyses.

## Conclusion

This study demonstrated that dietary EPA and DHA were effectively incorporated into serum phospholipid fractions in a dose-responsive manner and were well tolerated in adult maintenance dogs without evidence of adverse oxidative or inflammatory effects under the conditions tested. Collectively, these findings indicate that dogs may tolerate higher long-chain n-3 PUFA intakes than are currently recommended, particularly when diets provide adequate antioxidant support. These findings also support re-evaluation of current recommendations for EPA and DHA intake in dogs and warrant further investigation into the optimal dose of long-chain n-3 PUFA across different physiological and clinical contexts. Future work should also examine whether these responses translate to functional benefits, particularly in outcomes such as cognitive function and neuromuscular performance.

## Supplementary Material

skag257_Supplementary_Data

## Data Availability

The data that support the findings of this study are available from the corresponding author upon personal request.

## Supplementary data

*Conflict of interest statement*. Anna Kate Shoveller has received honoraria and research funding from various pet food manufacturers and ingredient suppliers, in addition to provincial and federal granting agencies. It declares that she serves on the Scientific Boards of Trouw Nutrition, Real Cat Science, and Champion Petfoods. Jennifer Saunders-Blades and Janelle Kelly were/are employed by the industry partner, Champion Petfoods Holding Inc., during the conduct of this trial, and no other authors have conflicts of interest relevant to this work.

## Abbreviations

AA: arachidonic acid
AAFCO: Association of American Feed Control Officials
ALA: α-linolenic acid
ANSA: 1-amino-2-naphthol-4-sulfonic acid
AOCS: American Oil Chemists’ Society
CCAC: Canadian Council on Animal Care
CCL2: CC motif chemokine ligand 2
CXCL1: C-X-C motif chemokine ligand 1
CXCL8: C-X-C motif chemokine ligand 8
CXCL10: C-X-C motif chemokine ligand 10
DBS: dried blood spot
DG: dosage group
DGLA: dihomo-γ-linolenic acid
DHA: docosahexaenoic acid
DPA: docosapentaenoic acid
ELISA: enzyme-linked immunosorbent assay
EPA: eicosapentaenoic acid
FA: fatty acid
FAME: fatty acid methyl ester
FID: flame ionization detector
GC: gas chromatography
GM-CSF: granulocyte-macrophage colony-stimulating factor
HPTLC: high-performance thinlayer chromatography
IFN-γ: interferon-γ
IL: interleukin
IP-10: interferon-γ-induced protein 10
KC-like: keratinocyte-derived chemokine-like
LA: linoleic acid
LCMS/MS: liquid chromatography–tandem mass spectrometry
LysoPC: lysophosphatidylcholine
MDA: malondialdehyde
MCP-1: monocyte chemoattractant protein 1
MRM: multiple reaction monitoring
MUFA: monounsaturated fatty acid
NRC: National Research Council
NSERC: Natural Sciences and Engineering Research Council of Canada
PC: phosphatidylcholine
PE: phosphatidylethanolamine
PGE2: prostaglandin E2
PUFA: polyunsaturated fatty acid
RA: recommended allowance
SFA: saturated fatty acid
SM: sphingomyelin
SUL: safe upper limit
TLC: thin-layer chromatography
TNF-α: tumor necrosis factor-α
