# Supplementary material for: Revisiting eicosapentaenoic acid and docosahexaenoic acid recommendations for healthy Siberian Huskies at maintenance: greater dietary EPA and DHA increase plasma lipid incorporation and reduce α-tocopherol concentrations
Source: J Anim Sci. 2026 Aug 13;104:skag257. doi: 10.1093/jas/skag257 (PMC13561521; doi:10.1093/jas/skag257)
Supplement: Supplementary file 1 [file skag257_supplementary_data.docx]

## **Revisiting Eicosapentaenoic Acid and Docosahexaenoic Acid Recommendations for Adult Dogs at Maintenance: Higher Doses Enhance Lipid Incorporation but Decrease Antioxidant Status**

Benjamin. D. Fox ^*^, Fumiko Imai ^*^, Sydney Banton ^*^, Alexandra Rankovic ^*^, Sarah MacDonald Murray ^*^, Lindsey Rummel^*^, David W.L. Ma ^‡^, Lyn M. Hillyer ^‡^, Jennifer Saunders-Blades ^†^, Janelle Kelly ^†^, James R. Templeman ^‡^, Lindsay E. Robinson ^‡^, Anna K. Shoveller^*§^

*Journal of Animal Science*

(Supplementary Materials S1, S2, S3, S4, S5, S6)

**Supplementary Table S1.** Proximate composition of the experimental diet on a dry-matter basis.

| **Nutrient contents** | **Analyzed content** | |
| --- | --- | --- |
| Metabolizable energy, kcal/kg (calculated) ^1^ | | 3848.85 |
| Dry matter, % | 100.00 | |
| Crude protein, % | 43.40 | |
| Crude fibre, % | 3.28 | |
| Nitrogen-free extract (NFE, calculated) ^2^ | 30.01 | |
| Ash, % | 8.26 | |
| Crude fat, % | 15.05 | |
| Fatty acids, % ^3^ |  | |
| 14:0 | 0.08 | |
| 15:0 | 0.01 | |
| 16:0 | 2.27 | |
| 18:0 | 0.72 | |
| 19:0 | <0.02 | |
| 20:0 | 0.02 | |
| 22:0 | <0.02 | |
| 23:0 | <0.02 | |
| 24:0 | <0.02 | |
| 16:1n7 | 0.47 | |
| 18:1n7 | 2.19 | |
| 18:1n9 | 5.02 | |
| 20:1n9 | 0.07 | |
| 22:1n9 | <0.02 | |
| 24:1n9 | <0.02 | |
| 18:2n6 (LA) | 3.56 | |
| 18:3n6 | <0.02 | |
| 20:2n6 | 0.02 | |
| 20:3n6 (DGLA) | 0.02 | |
| 20:4n6 (AA) | 0.13 | |
| 22:2n6 | <0.02 | |
| 22:4n6 | 0.04 | |
| 22:5n6 | <0.02 | |
| 18:3n3 (ALA) | 0.25 | |
| 18:4n3 | <0.02 | |
| 20:5n3 (EPA) | 0.03 | |
| 22:5n3 | 0.03 | |
| 22:6n3 (DHA) | 0.04 | |
| Saturated fatty acids (SFA) | 3.12 | |
| Monounsaturated fatty acids (MUFA) | 7.75 | |
| Total n-6 | 3.77 | |
| EPA+DHA | 0.07 | |
| Total n-3 | 0.37 | |
| Polyunsaturated fatty acids (PUFA) | 4.12 | |
| Minerals and vitamins |  | |
| Vitamin A, IU/kg | 7696.51 | |
| α-Tocopherol, IU/kg | 1888.65 | |

^1^ Calculated metabolizable energy based on modified Atwater values.

^2^ NFE= 100 - (moisture + protein + fat + fibre + ash).

^3^ Fatty acids were analyzed by gas liquid chromatography at the University of Guelph.

**Supplementary Table S2.** Feed intake (FI) and body weight (BW) from week 1 to week 8 in dogs (n=27) assigned to experimental dose groups (DG) of eicosapentaenoic acid (EPA) and docosahexaenoic acid (DHA) [LOW (0.03 g/kg BW⁰·⁷⁵), MOD (0.45 g/kg BW⁰·⁷⁵, and HIGH (0.71 g/kg BW⁰·⁷⁵)] over 8 weeks, with corresponding p-values for main (DG, week) and interaction (DG × week) effects. Different superscripts (^a,b,c,d,e,f^) within the same row indicate statistical significance (P ≤ 0.05).

| DG | Week | | | | | | | | | P-value | | |
| --- | --- | --- | --- | --- | --- | --- | --- | --- | --- | --- | --- | --- |
|  | 1 | 2 | 3 | | 4 | 5 | 6 | 7 | 8 | DG | Week | DG x Week |
| **Feed intake, g/d** |  |  | |  |  |  |  |  |  |  |  |  |
| Overall mean (n=27) | 262.8±11.6^e^ | 244.0±10.8^f^ | 244.5±10.8^f^ | | 261.8±12.3^e^ | 286.7±11.8^d^ | 333.8±15.4^c^ | 417.1±16.2^b^ | 455.3±21.7^a^ | 0.123 | <0.001 | 0.744 |
| LOW (n=9) | 296.0±20.0 | 275.4±18.8 | 275.4±18.6 | | 297.5±21.3 | 323.3±20.5 | 380.0±26.7 | 471.3±28.1 | 496.5±37.6 |  |  |  |
| MOD (n=9) | 251.2±20.0 | 233.8±18.8 | 233.8±18.6 | | 246.4±21.3 | 271.1±20.5 | 320.2±26.7 | 391.7±28.1 | 436.9±37.6 |  |  |  |
| HIGH (n=9) | 241.1±20.0 | 222.8±18.8 | 224.4±18.6 | | 241.4±21.3 | 265.6±20.5 | 301.3±26.8 | 388.3±28.1 | 432.4±37.6 |  |  |  |
|  |  |  |  | |  |  |  |  |  |  |  |  |
| **Body weight, kg** |  |  |  | |  |  |  |  |  |  |  |  |
| Overall mean (n=27) | 21.5±0.6 ^a^ | 21.1±0.6 ^b^ | 21.1±0.6 ^bc^ | | 20.9±0.6 ^bc^ | 21.0±0.5 ^bc^ | 20.8±0.6 ^c^ | 21.1±0.6 ^b^ | 21.2±0.6 ^b^ | 0.369 | <0.001 | 0.286 |
| LOW (n=9) | 21.3±1.1 | 21.2±1.0 | 20.8±1.0 | | 20.7±1.0 | 20.7±0.9 | 20.5±1.0 | 20.9±1.0 | 21.0±1.0 |  |  |  |
| MOD (n=9) | 22.5±1.1 | 22.4±1.0 | 22.0±1.0 | | 21.9±1.0 | 21.9±0.9 | 22.0±1.0 | 22.2±1.0 | 22.1±1.0 |  |  |  |
| HIGH (n=9) | 20.7±1.1 | 20.9±1.0 | 20.5±1.0 | | 20.3±1.0 | 20.3±0.9 | 19.9±1.0 | 20.2±1.0 | 20.5±1.0 |  |  |  |
|  |  |  |  | |  |  |  |  |  |  |  |  |

**Supplementary Table S3.** Least squares means (LSMeans ± SEM) for serum fatty acids (%) in phosphatidylcholine (PC) across weeks (0, 4, and 8) and dose groups (DG) of eicosapentaenoic acid (EPA) and docosahexaenoic acid (DHA) [LOW (0.03 g/kg BW⁰·⁷⁵), MOD (0.45 g/kg BW⁰·⁷⁵), and HIGH (0.71 g/kg BW⁰·⁷⁵)] over 8 weeks, with corresponding p-values for main (DG, week) and interaction (DG × week) effects. Week 0 (pre-treatment baseline) was used as a covariate, and Mean values represent LSMeans averaged over Weeks 4 and 8 (baseline-adjusted). Different superscript letters (^a,b^) indicate statistically significant differences among DGs (P ≤ 0.05), and different subscript letters (_a,b_) indicate statistically significant differences in DG x Week interaction (P ≤ 0.05).

| Fatty acid in PC (%) |  | Week | | | |  | P-value | | |
| --- | --- | --- | --- | --- | --- | --- | --- | --- | --- |
|  |  | 0 | 4 | 8 | Mean |  | DG | Week | DGxWeek |
| 14:0 | LOW | 0.18±0.05 | 0.18 ± 0.04 | 0.17 ± 0.04 | 0.18 ± 0.03 |  | 0.29 | 0.86 | 0.97 |
|  | MOD | 0.24±0.05 | 0.25 ± 0.04 | 0.23 ± 0.04 | 0.24 ± 0.03 |  |  |  |  |
|  | HIGH | 0.33±0.05 | 0.20 ± 0.04 | 0.20 ± 0.04 | 0.20 ± 0.03 |  |  |  |  |
|  |  |  |  |  |  |  |  |  |  |
| 15:0 | LOW | 0.22±0.06 | 0.14 ± 0.02 | 0.13 ± 0.02 | 0.14 ± 0.01 |  | 0.81 | 0.44 | 0.77 |
|  | MOD | 0.27±0.06 | 0.15 ± 0.02 | 0.15 ± 0.02 | 0.15 ± 0.01 |  |  |  |  |
|  | HIGH | 0.26±0.06 | 0.15 ± 0.02 | 0.13 ± 0.02 | 0.14 ± 0.01 |  |  |  |  |
|  |  |  |  |  |  |  |  |  |  |
| 16:0 | LOW | 16.25 ± 0.37 | 16.09 ± 0.35 | 15.71 ± 0.35 | 15.90 ± 0.30 |  | 0.09 | 0.49 | 0.64 |
|  | MOD | 16.42 ± 0.37 | 14.90 ± 0.35 | 15.01 ± 0.35 | 14.96 ± 0.30 |  |  |  |  |
|  | HIGH | 16.25 ± 0.37 | 15.25 ± 0.35 | 15.08 ± 0.35 | 15.17 ± 0.30 |  |  |  |  |
|  |  |  |  |  |  |  |  |  |  |
| 18:0 | LOW | 24.13 ± 0.49 | 24.78 ± 0.45 | 25.05 ± 0.45 | 24.91 ± 0.39 |  | 0.54 | 0.01 | 0.02 |
|  | MOD | 24.40 ± 0.49 | 26.30 ± 0.45 | 24.77 ± 0.45 | 25.53 ± 0.40 |  |  |  |  |
|  | HIGH | 23.85 ± 0.49 | 25.55 ± 0.45 | 24.76 ± 0.45 | 25.16 ± 0.40 |  |  |  |  |
|  |  |  |  |  |  |  |  |  |  |
| 19:0 | LOW | 0.52 ± 0.13 | 0.23 ± 0.02 | 0.26 ± 0.02 | 0.25 ± 0.02 |  | 0.49 | 0.26 | 0.02 |
|  | MOD | 0.57 ± 0.13 | 0.28 ± 0.02 | 0.27 ± 0.02 | 0.28 ± 0.02 |  |  |  |  |
|  | HIGH | 0.44 ± 0.13 | 0.29 ± 0.02 | 0.22 ± 0.02 | 0.25 ± 0.02 |  |  |  |  |
|  |  |  |  |  |  |  |  |  |  |
| 20:0 | LOW | 0.09 ± 0.02 | 0.09 ± 0.01 | 0.11 ± 0.01 | 0.10 ± 0.01^b^ |  | 0.02 | 0.53 | 0.29 |
|  | MOD | 0.06 ± 0.02 | 0.10 ± 0.01 | 0.12 ± 0.01 | 0.11 ± 0.01^ab^ |  |  |  |  |
|  | HIGH | 0.08 ± 0.02 | 0.14 ± 0.01 | 0.12 ± 0.01 | 0.13 ± 0.01^a^ |  |  |  |  |
|  |  |  |  |  |  |  |  |  |  |
| 22:0 | LOW | 0.07 ± 0.03 | 0.07 ± 0.02 | 0.11 ± 0.02 | 0.09 ± 0.01 |  | 0.10 | 0.26 | 0.90 |
|  | MOD | 0.06 ± 0.03 | 0.08 ± 0.02 | 0.10 ± 0.02 | 0.09 ± 0.01 |  |  |  |  |
|  | HIGH | 0.10 ± 0.03 | 0.12 ± 0.02 | 0.14 ± 0.02 | 0.13 ± 0.01 |  |  |  |  |
|  |  |  |  |  |  |  |  |  |  |
| 23:0 | LOW | ND | 0.03 ± 0.01 | 0.00 ± 0.01 | 0.01 ± 0.01 |  | 0.74 | <0.01 | 0.74 |
|  | MOD | ND | 0.02 ± 0.01 | 0.00 ± 0.01 | 0.01 ± 0.01 |  |  |  |  |
|  | HIGH | ND | 0.04 ± 0.01 | 0.00 ± 0.01 | 0.012 ± 0.01 |  |  |  |  |
|  |  |  |  |  |  |  |  |  |  |
| 24:0 | LOW | 0.22 ± 0.07 | 0.10 ± 0.04 | 0.13 ± 0.04 | 0.11 ± 0.03^ab^ |  | 0.05 | 0.58 | 0.09 |
|  | MOD | 0.17 ± 0.07 | 0.10 ± 0.04 | 0.02 ± 0.04 | 0.06 ± 0.03^b^ |  |  |  |  |
|  | HIGH | 0.10 ± 0.07 | 0.12 ± 0.04 | 0.23 ± 0.04 | 0.18 ± 0.03^a^ |  |  |  |  |
|  |  |  |  |  |  |  |  |  |  |
| Total SFA | LOW | 42.24 ± 0.39 | 41.71 ± 0.39 | 42.14 ± 0.39 | 41.93 ± 0.29 |  | 0.56 | 0.67 | 0.45 |
|  | MOD | 42.53 ± 0.39 | 42.26 ± 0.39 | 41.78 ± 0.39 | 42.02 ± 0.29 |  |  |  |  |
|  | HIGH | 42.13 ± 0.39 | 41.77 ± 0.39 | 41.42 ± 0.39 | 41.60 ± 0.29 |  |  |  |  |
|  |  |  |  |  |  |  |  |  |  |
| 16:1n7 | LOW | 0.46 ± 0.03 | 0.42 ± 0.03 | 0.41 ± 0.03 | 0.41 ± 0.03 |  | 0.67 | 0.25 | 0.64 |
|  | MOD | 0.47 ± 0.03 | 0.39 ± 0.03 | 0.38 ± 0.03 | 0.38 ± 0.03 |  |  |  |  |
|  | HIGH | 0.49 ± 0.03 | 0.41 ± 0.03 | 0.37 ± 0.03 | 0.39 ± 0.03 |  |  |  |  |
|  |  |  |  |  |  |  |  |  |  |
| 18:1n7 | LOW | 2.33 ± 0.07 | 2.20 ± 0.09 | 1.99 ± 0.09 | 2.10 ± 0.07 |  | 0.29 | 0.01 | 0.86 |
|  | MOD | 2.50 ± 0.07 | 2.26 ± 0.09 | 2.10 ± 0.09 | 2.18 ± 0.07 |  |  |  |  |
|  | HIGH | 2.48 ± 0.07 | 2.33 ± 0.09 | 2.20 ± 0.09 | 2.27 ± 0.07 |  |  |  |  |
|  |  |  |  |  |  |  |  |  |  |
| 18:1n9 | LOW | 7.25 ± 0.18 | 6.63 ± 0.16 | 6.78 ± 0.16 | 6.71 ± 0.14 |  | 0.32 | 0.01 | 0.52 |
|  | MOD | 7.13 ± 0.18 | 6.28 ± 0.16 | 6.71 ± 0.16 | 6.49 ± 0.14 |  |  |  |  |
|  | HIGH | 7.24 ± 0.18 | 6.61 ± 0.16 | 6.95 ± 0.16 | 6.78 ± 0.14 |  |  |  |  |
|  |  |  |  |  |  |  |  |  |  |
| 20:1n9 | LOW | 0.37 ± 0.05 | 0.29 ± 0.03 | 0.27 ± 0.03 | 0.28 ± 0.02 |  | 0.18 | 0.31 | 0.86 |
|  | MOD | 0.33 ± 0.05 | 0.33 ± 0.03 | 0.31 ± 0.03 | 0.32 ± 0.02 |  |  |  |  |
|  | HIGH | 0.34 ± 0.05 | 0.36 ± 0.03 | 0.30 ± 0.03 | 0.33 ± 0.02 |  |  |  |  |
|  |  |  |  |  |  |  |  |  |  |
| 22:1n9 | LOW | 0.96 ± 0.21 | 0.30 ± 0.07 | 0.27 ± 0.07 | 0.29 ± 0.04^ab^ |  | 0.02 | 0.45 | 0.39 |
|  | MOD | 0.81 ± 0.21 | 0.28 ± 0.07 | 0.11 ± 0.07 | 0.20 ± 0.04^b^ |  |  |  |  |
|  | HIGH | 0.87 ± 0.21 | 0.35 ± 0.07 | 0.39 ± 0.07 | 0.37 ± 0.04^a^ |  |  |  |  |
|  |  |  |  |  |  |  |  |  |  |
| 24:1n9 | LOW | 0.33 ± 0.09 | 0.12 ± 0.05 | 0.18 ± 0.05 | 3.53 ± 0.84 |  | 0.41 | 0.88 | 0.74 |
|  | MOD | 0.26 ± 0.09 | 0.11 ± 0.05 | 0.14 ± 0.05 | 4.84 ± 0.84 |  |  |  |  |
|  | HIGH | 0.09 ± 0.09 | 0.21 ± 0.06 | 0.16 ± 0.06 | 5.02 ± 0.84 |  |  |  |  |
|  |  |  |  |  |  |  |  |  |  |
| Total MUFA | LOW | 11.69 ± 0.36 | 9.93 ± 0.25 | 9.88 ± 0.25 | 9.90 ± 0.19 |  | 0.06 | 0.78 | 0.93 |
|  | MOD | 11.51 ± 0.36 | 9.64 ± 0.25 | 9.74 ± 0.25 | 9.69 ± 0.19 |  |  |  |  |
|  | HIGH | 11.51 ± 0.36 | 10.30 ± 0.25 | 10.41 ± 0.25 | 10.36 ± 0.19 |  |  |  |  |
|  |  |  |  |  |  |  |  |  |  |
| 18:2n6 (LA) | LOW | 20.09 ± 0.41 | 20.04 ± 0.79 | 21.04 ± 0.79 | 20.54 ± 0.69^a^ |  | <0.01 | <0.01 | 0.57 |
|  | MOD | 20.10 ± 0.41 | 15.57 ± 0.79 | 17.58 ± 0.79 | 16.58 ± 0.69^b^ |  |  |  |  |
|  | HIGH | 20.00 ± 0.41 | 15.73 ± 0.79 | 17.75 ± 0.79 | 16.74 ± 0.69^b^ |  |  |  |  |
|  |  |  |  |  |  |  |  |  |  |
| 20:3n6 | LOW | 0.54 ± 0.22 | 1.38 ± 0.11_a_ | 0.00 ± 0.11_d_ | 0.69 ± 0.08 |  | 0.34 | <0.01 | <0.01 |
|  | MOD | 0.41 ± 0.22 | 1.10 ± 0.11_ab_ | 0.30 ± 0.11_cd_ | 0.70 ± 0.08 |  |  |  |  |
|  | HIGH | 0.89 ± 0.22 | 1.05 ± 0.11_ab_ | 0.64 ± 0.11_bc_ | 0.84 ± 0.08 |  |  |  |  |
|  |  |  |  |  |  |  |  |  |  |
| 20:4n6 (AA) | LOW | 17.85 ± 0.49 | 19.13 ± 0.73 | 18.05 ± 0.73 | 18.59 ± 0.59^a^ |  | <0.01 | 0.30 | 0.28 |
|  | MOD | 18.28 ± 0.49 | 16.05 ± 0.73 | 14.89 ± 0.73 | 15.47 ± 0.59^b^ |  |  |  |  |
|  | HIGH | 18.11 ± 0.49 | 14.16 ± 0.73 | 14.79 ± 0.73 | 14.47 ± 0.59^b^ |  |  |  |  |
|  |  |  |  |  |  |  |  |  |  |
| 22:4n6 | LOW | 0.56 ± 0.04 | 0.76 ± 0.08 | 0.72 ± 0.08 | 0.74 ± 0.07^a^ |  | <0.01 | 0.45 | 0.21 |
|  | MOD | 0.53 ± 0.04 | 0.28 ± 0.08 | 0.27 ± 0.08 | 0.27 ± 0.07^b^ |  |  |  |  |
|  | HIGH | 0.51 ± 0.04 | 0.27 ± 0.08 | 0.43 ± 0.08 | 0.35 ± 0.07^b^ |  |  |  |  |
|  |  |  |  |  |  |  |  |  |  |
| 22:5n6 | LOW | 0.27 ± 0.03 | 0.31 ± 0.03 | 0.25 ± 0.03 | 0.28 ± 0.02^a^ |  | <0.01 | 0.69 | 0.23 |
|  | MOD | 0.32 ± 0.03 | 0.16 ± 0.03 | 0.17 ± 0.03 | 0.16 ± 0.02^b^ |  |  |  |  |
|  | HIGH | 0.32 ± 0.03 | 0.12 ± 0.03 | 0.14 ± 0.03 | 0.13 ± 0.02^b^ |  |  |  |  |
|  |  |  |  |  |  |  |  |  |  |
| Total n-6 | LOW | 39.97 ± 0.37 | 42.18 ± 1.17 | 40.57 ± 1.17 | 41.37 ± 0.94^a^ |  | <0.01 | 0.69 | 0.14 |
|  | MOD | 40.40 ± 0.37 | 33.71 ± 1.16 | 33.87 ± 1.16 | 33.79 ± 0.93^b^ |  |  |  |  |
|  | HIGH | 40.56 ± 0.37 | 31.82 ± 1.16 | 34.24 ± 1.16 | 33.03 ± 0.94^b^ |  |  |  |  |
|  |  |  |  |  |  |  |  |  |  |
|  |  |  |  |  |  |  |  |  |  |
| 18:3n3 (ALA) | LOW | 0.31±0.05 | 0.29±0.02 | 0.32±0.02 | 0.30 ± 0.01 |  | 0.17 | 0.02 | 0.45 |
|  | MOD | 0.31±0.05 | 0.24±0.02 | 0.30±0.02 | 0.27 ± 0.01 |  |  |  |  |
|  | HIGH | 0.27±0.05 | 0.26±0.02 | 0.28±0.02 | 0.27 ± 0.01 |  |  |  |  |
|  |  |  |  |  |  |  |  |  |  |
| 20:3n3 | LOW | 0.10 ± 0.04 | 0.05 ± 0.02 | 0.05 ± 0.02 | 0.05 ± 0.01^ab^ |  | 0.04 | 0.85 | 0.32 |
|  | MOD | 0.02 ± 0.04 | 0.07 ± 0.02 | 0.10 ± 0.02 | 0.08 ± 0.01^a^ |  |  |  |  |
|  | HIGH | ND | 0.05 ± 0.02 | 0.03 ± 0.02 | 0.05 ± 0.01^b^ |  |  |  |  |
|  |  |  |  |  |  |  |  |  |  |
| 20:5n3 (EPA) | LOW | 0.90 ± 0.05 | 1.45 ± 0.90 | 2.77 ± 0.9 | 2.11 ± 0.76^b^ |  | <0.01 | 0.96 | 0.23 |
|  | MOD | 0.81 ± 0.05 | 7.26 ± 0.90 | 7.19 ± 0.90 | 7.22 ± 0.75^a^ |  |  |  |  |
|  | HIGH | 0.84 ± 0.05 | 8.67 ± 0.89 | 7.50 ± 0.89 | 8.08 ± 0.74^a^ |  |  |  |  |
|  |  |  |  |  |  |  |  |  |  |
| 22:5n3 | LOW | 1.73 ± 0.09 | 1.77 ± 0.14 | 1.81 ± 0.14 | 1.80 ± 0.12 ^b^ |  | 0.03 | 0.06 | 0.18 |
|  | MOD | 1.76 ± 0.09 | 2.03 ± 0.14 | 2.36 ± 0.14 | 2.20 ± 0.12 ^a^ |  |  |  |  |
|  | HIGH | 1.54 ± 0.09 | 2.21 ± 0.14 | 2.26 ± 0.14 | 2.23 ± 0.13 ^a^ |  |  |  |  |
|  |  |  |  |  |  |  |  |  |  |
| 22:6n3 (DHA) | LOW | 2.78 ± 0.14 | 2.44 ± 0.32 | 2.15 ± 0.32 | 2.29 ± 0.27^b^ |  | <0.01 | <0.01 | 0.10 |
|  | MOD | 2.46 ± 0.14 | 4.43 ± 0.32 | 3.94 ± 0.32 | 4.19 ± 0.27^a^ |  |  |  |  |
|  | HIGH | 2.65 ± 0.14 | 4.83 ± 0.31 | 3.57 ± 0.31 | 4.20 ± 0.27^a^ |  |  |  |  |
|  |  |  |  |  |  |  |  |  |  |
| EPA+DHA | LOW | 3.68 ± 0.16 | 4.06 ± 1.14 | 5.09 ± 1.14 | 4.58 ± 0.95^b^ |  | <0.01 | 0.38 | 0.17 |
|  | MOD | 3.27 ± 0.16 | 11.47 ± 1.14 | 10.90 ± 1.14 | 11.19 ± 0.95^a^ |  |  |  |  |
|  | HIGH | 3.50 ± 0.16 | 13.55 ± 1.12 | 11.11 ± 1.12 | 12.33 ± 0.92^a^ |  |  |  |  |
|  |  |  |  |  |  |  |  |  |  |
| Total n-3 | LOW | 5.81 ± 0.21 | 6.30 ± 1.20 | 7.40 ± 1.20 | 6.85 ± 1.00^b^ |  | <0.01 | 0.54 | 0.19 |
|  | MOD | 5.35 ± 0.21 | 13.98 ± 1.17 | 13.85 ± 1.17 | 13.92 ± 0.97^a^ |  |  |  |  |
|  | HIGH | 5.31 ± 0.21 | 15.76 ± 1.18 | 13.36 ± 1.18 | 14.57 ± 0.97^a^ |  |  |  |  |
|  |  |  |  |  |  |  |  |  |  |
| Total PUFA | LOW | 45.77 ± 0.41 | 48.19 ± 0.40 | 47.69 ± 0.40 | 47.94 ± 0.25 |  | 0.81 | 0.69 | 0.79 |
|  | MOD | 45.75 ± 0.41 | 47.85 ± 0.40 | 47.89 ± 0.40 | 47.87 ± 0.25 |  |  |  |  |
|  | HIGH | 45.87 ± 0.41 | 47.71 ± 0.40 | 47.73 ± 0.40 | 47.72 ± 0.25 |  |  |  |  |
|  |  |  |  |  |  |  |  |  |  |
| n-6: n-3 | LOW | 7.02 ± 0.31 | 7.16 ± 0.74 | 7.87 ± 0.74 | 7.51 ± 0.58^a^ |  | <0.01 | 0.14 | 0.49 |
|  | MOD | 7.62 ± 0.31 | 2.48 ± 0.72 | 2.56 ± 0.72 | 2.52 ± 0.57^b^ |  |  |  |  |
|  | HIGH | 7.71 ± 0.31 | 2.13 ± 0.72 | 3.74 ± 0.72 | 2.94 ± 0.57^b^ |  |  |  |  |
|  |  |  |  |  |  |  |  |  |  |
| AA: EPA+DHA | LOW | 4.96 ± 0.45 | 5.13 ± 0.66 | 6.07 ± 0.66 | 5.60 ± 0.51^a^ |  | <0.01 | 0.10 | 0.44 |
|  | MOD | 5.14 ± 0.45 | 1.62 ± 0.66 | 1.63 ± 0.66 | 1.63 ± 0.51^b^ |  |  |  |  |
|  | HIGH | 5.24 ± 0.45 | 1.06 ± 0.65 | 2.62 ± 0.65 | 1.84 ± 0.49^b^ |  |  |  |  |

^1^Mean indicates week data pooled across weeks

^a,b,c^Values in columns with different superscripts differ across time for a particular treatment group (*P* < 0.05)

**Supplementary Table S4.** Least squares means (LSMeans ± SEM) for serum fatty acids (%) in lysophosphatidylcholine (lysoPC) of dogs (n = 27) across weeks (0, 4, and 8) and dose groups (DG) of eicosapentaenoic acid (EPA) and docosahexaenoic acid (DHA) [LOW (0.03 g/kg BW⁰·⁷⁵), MOD (0.45 g/kg BW⁰·⁷⁵), and HIGH (0.71 g/kg BW⁰·⁷⁵)] over 8 weeks, with corresponding p-values for main (DG, week) and interaction (DG × week) effects. Week 0 (pre-treatment baseline) was used as a covariate, and Mean values represent LSMeans averaged over Weeks 4 and 8 (baseline-adjusted). Different superscript letters (^a,b^) indicate statistically significant differences among DGs (P ≤ 0.05).

| Fatty acid in LysoPC (%) |  | DG | | |  |  | P-value | | |
| --- | --- | --- | --- | --- | --- | --- | --- | --- | --- |
|  |  | 0 | 4 | 8 | Mean |  | DG | Week | DGxWeek |
| 14:0 | LOW | 0.87 ± 0.21 | 1.07 ± 0.26 | 1.18 ± 0.26 | 1.12 ± 0.15 |  | 0.37 | 0.94 | 0.93 |
|  | MOD | 0.81 ± 0.21 | 1.36 ± 0.26 | 1.25 ± 0.26 | 1.31 ± 0.15 |  |  |  |  |
|  | HIGH | 1.07 ± 0.21 | 1.41 ± 0.26 | 1.47 ± 0.26 | 1.44 ± 0.16 |  |  |  |  |
|  |  |  |  |  |  |  |  |  |  |
| 15:0 | LOW | 1.45 ± 0.42 | 0.47 ± 0.15 | 0.63 ± 0.15 | 0.55 ± 0.12 |  | 0.18 | 0.78 | 0.71 |
|  | MOD | 1.42 ± 0.42 | 0.28 ± 0.15 | 0.28 ± 0.15 | 0.28 ± 0.12 |  |  |  |  |
|  | HIGH | 1.42 ± 1.42 | 0.61 ± 0.15 | 0.55 ± 0.15 | 0.58 ± 0.12 |  |  |  |  |
|  |  |  |  |  |  |  |  |  |  |
| 16:0 | LOW | 31.72 ± 1.28 | 35.38 ± 0.98 | 33.09 ± 0.98 | 34.23 ± 0.69 |  | 0.17 | 0.01 | 0.93 |
|  | MOD | 31.90 ± 1.28 | 36.65 ± 0.98 | 33.85 ± 0.98 | 35.25 ± 0.69 |  |  |  |  |
|  | HIGH | 31.80 ± 1.28 | 34.37 ± 0.98 | 32.32 ± 0.98 | 33.34 ± 0.69 |  |  |  |  |
|  |  |  |  |  |  |  |  |  |  |
| 18:0 | LOW | 22.62 ± 0.95 | 24.06 ± 0.95 | 23.95 ± 0.95 | 24.01 ± 0.75 |  | 0.18 | 0.40 | 0.37 |
|  | MOD | 22.04 ± 0.95 | 26.92 ± 0.96 | 24.98 ± 0.96 | 25.95 ± 0.75 |  |  |  |  |
|  | HIGH | 22.78 ± 0.95 | 24.23 ± 0.96 | 24.55 ± 0.96 | 24.39 ± 0.75 |  |  |  |  |
|  |  |  |  |  |  |  |  |  |  |
| 19:0 | LOW | 3.31 ± 1.06 | 1.09 ± 0.17 | 0.76 ± 0.17 | 0.92 ± 0.15 |  | 0.47 | <0.01 | 0.09 |
|  | MOD | 3.07 ± 1.06 | 1.39 ± 0.17 | 0.58 ± 0.17 | 0.98 ± 0.15 |  |  |  |  |
|  | HIGH | 2.74 ± 1.06 | 1.29 ± 0.17 | 1.05 ± 0.17 | 1.17 ± 0.15 |  |  |  |  |
|  |  |  |  |  |  |  |  |  |  |
| 20:0 | LOW | 0.27 ± 0.19 | 0.81 ± 0.13 | 0.43 ± 0.13 | 0.62 ± 0.10 |  | 0.90 | <0.01 | 0.12 |
|  | MOD | 0.55 ± 0.19 | 0.62 ± 0.13 | 0.53 ± 0.13 | 0.57 ± 0.10 |  |  |  |  |
|  | HIGH | 0.31 ± 0.19 | 0.93 ± 0.13 | 0.35 ± 0.13 | 0.64 ± 0.10 |  |  |  |  |
|  |  |  |  |  |  |  |  |  |  |
| 22:0 | LOW | 0.30 ± 0.17 | 0.95 ± 0.17 | 0.58 ± 0.17 | 0.76 ± 0.12 |  | 0.29 | 0.20 | 0.13 |
|  | MOD | 0.57 ± 0.17 | 0.39 ± 0.17 | 0.62 ± 0.17 | 0.50 ± 0.12 |  |  |  |  |
|  | HIGH | 0.13 ± 0.17 | 0.75 ± 0.17 | 0.36 ± 0.17 | 0.55 ± 0.12 |  |  |  |  |
|  |  |  |  |  |  |  |  |  |  |
| 23:0 | LOW | 0.00 ± 0.00 | 0.62 ± 0.10 | 0.00 ± 0.10 | 0.31 ± 0.07 |  | 0.09 | <0.01 | 0.09 |
|  | MOD | 0.00 ± 0.00 | 0.18 ± 0.10 | 0.00 ± 0.10 | 0.09 ± 0.07 |  |  |  |  |
|  | HIGH | 0.00 ± 0.00 | 0.35 ± 0.10 | 0.00 ± 0.10 | 0.17 ± 0.07 |  |  |  |  |
|  |  |  |  |  |  |  |  |  |  |
| 24:0 | LOW | 0.16 ± 0.18 | 0.86 ± 0.16 | 0.37 ± 0.16 | 0.62 ± 0.11 |  | 0.74 | <0.01 | 0.82 |
|  | MOD | 0.49 ± 0.18 | 0.76 ± 0.16 | 0.46 ± 0.16 | 0.61 ± 0.12 |  |  |  |  |
|  | HIGH | 0.12 ± 0.18 | 0.69 ± 0.16 | 0.31 ± 0.16 | 0.50 ± 0.12 |  |  |  |  |
|  |  |  |  |  |  |  |  |  |  |
| Total SFA | LOW | 62.71 ± 1.94 | 65.37 ± 1.75 | 63.67 ± 1.75 | 64.52 ± 1.27 |  | 0.40 | 0.12 | 0.70 |
|  | MOD | 61.96 ± 1.94 | 68.41 ± 1.75 | 64.60 ± 1.75 | 66.51 ± 1.28 |  |  |  |  |
|  | HIGH | 64.89 ± 1.94 | 64.71 ± 1.76 | 63.62 ± 1.76 | 64.16 ± 1.29 |  |  |  |  |
|  |  |  |  |  |  |  |  |  |  |
| 16:1n7 | LOW | 0.28 ± 0.17 | 0.58 ± 0.25 | 1.15 ± 0.25 | 0.87 ± 0.16 |  | 0.36 | 0.26 | 0.60 |
|  | MOD | 0.32 ± 0.17 | 0.59 ± 0.25 | 0.63 ± 0.25 | 0.61 ± 0.16 |  |  |  |  |
|  | HIGH | 0.58 ± 0.17 | 0.47 ± 0.25 | 0.63 ± 0.25 | 0.55 ± 0.16 |  |  |  |  |
|  |  |  |  |  |  |  |  |  |  |
| 18:1n7 | LOW | 2.40 ± 0.29 | 1.62 ± 0.12 | 1.68 ± 0.12 | 1.65 ± 0.10 |  | 0.63 | 0.27 | 0.44 |
|  | MOD | 2.00 ± 0.29 | 1.78 ± 0.12 | 1.77 ± 0.12 | 1.77 ± 0.10 |  |  |  |  |
|  | HIGH | 2.20 ± 0.29 | 1.64 ± 0.12 | 1.87 ± 0.12 | 1.76 ± 0.10 |  |  |  |  |
|  |  |  |  |  |  |  |  |  |  |
| 18:1n9 | LOW | 7.60 ± 0.43 | 6.44 ± 0.29 | 7.40 ± 0.29 | 6.92 ± 0.23 |  | 0.97 | 0.02 | 0.25 |
|  | MOD | 6.67 ± 0.43 | 6.92 ± 0.30 | 7.09 ± 0.30 | 7.01 ± 0.25 |  |  |  |  |
|  | HIGH | 8.03 ± 0.43 | 6.78 ± 0.30 | 7.14 ± 0.30 | 6.96 ± 0.24 |  |  |  |  |
|  |  |  |  |  |  |  |  |  |  |
| 20:1n9 | LOW | 0.00 ± 0.00 | 0.02 ± 0.04 | 0.00 ± 0.04 | 0.07 ± 0.03 |  | 0.42 | 0.04 | 0.42 |
|  | MOD | 0.00 ± 0.00 | 0.07 ± 0.04 | 0.00 ± 0.04 | 0.03 ± 0.03 |  |  |  |  |
|  | HIGH | 0.00 ± 0.00 | 0.14 ± 0.04 | 0.00 ± 0.04 | 0.07 ± 0.03 |  |  |  |  |
|  |  |  |  |  |  |  |  |  |  |
| 22:1n9 | LOW | 9.54 ± 2.70 | 2.69 ± 1.59 | 3.81 ± 1.59 | 3.25 ± 1.18 |  | 0.33 | 0.13 | 0.81 |
|  | MOD | 13.29 ± 2.70 | 2.87 ± 1.62 | 5.88 ± 1.62 | 4.38 ± 1.22 |  |  |  |  |
|  | HIGH | 6.49 ± 2.70 | 5.04 ± 1.61 | 6.64 ± 1.61 | 5.84 ± 1.22 |  |  |  |  |
|  |  |  |  |  |  |  |  |  |  |
| 24:1n9 | LOW | 0.94 ± 0.39 | 2.10 ± 0.38 | 1.40 ± 0.38 | 1.75 ± 0.27 |  | 0.46 | 0.23 | 0.16 |
|  | MOD | 1.49 ± 0.39 | 1.03 ± 0.39 | 1.52 ± 0.39 | 1.28 ± 0.28 |  |  |  |  |
|  | HIGH | 0.34 ± 0.39 | 1.88 ± 0.39 | 0.94 ± 0.39 | 1.41 ± 0.28 |  |  |  |  |
|  |  |  |  |  |  |  |  |  |  |
| Total MUFA | LOW | 20.76 ± 2.24 | 13.44 ± 1.61 | 15.43 ± 1.61 | 14.44 ± 1.17 |  | 0.38 | 0.09 | 0.74 |
|  | MOD | 23.77 ± 2.24 | 13.10 ± 1.64 | 16.73 ± 1.64 | 14.91 ± 1.21 |  |  |  |  |
|  | HIGH | 17.65 ± 2.24 | 16.13 ± 1.64 | 17.39 ± 1.64 | 16.76 ± 1.21 |  |  |  |  |
|  |  |  |  |  |  |  |  |  |  |
| 18:2n6 (LA) | LOW | 9.64 ± 0.78 | 10.72 ± 0.76 | 12.32 ± 0.76 | 11.52 ± 0.63^a^ |  | 0.02 | <0.01 | 0.99 |
|  | MOD | 8.32 ± 0.78 | 8.85 ± 0.78 | 10.25 ± 0.78 | 9.55 ± 0.64^ab^ |  |  |  |  |
|  | HIGH | 9.85 ± 0.78 | 8.30 ± 0.77 | 9.76 ± 0.77 | 9.03 ± 0.63^b^ |  |  |  |  |
|  |  |  |  |  |  |  |  |  |  |
| 20:3n6 | LOW | 0.18 ± 0.14 | 0.72 ± 0.11 | 0.16 ± 0.11 | 0.44 ± 0.07^a^ |  | 0.03 | <0.01 | 0.19 |
|  | MOD | 0.36 ± 0.14 | 0.35 ± 0.11 | 0.00 ± 0.11 | 0.17 ± 0.07^b^ |  |  |  |  |
|  | HIGH | 0.17 ± 0.14 | 0.40 ± 0.11 | 0.28 ± 0.11 | 0.34 ± 0.07^ab^ |  |  |  |  |
|  |  |  |  |  |  |  |  |  |  |
| 20:4n6 (AA) | LOW | 4.23 ± 0.33 | 4.71 ± 0.33 | 4.50 ± 0.33 | 4.61 ± 0.22^a^ |  | <0.01 | 0.26 | 0.96 |
|  | MOD | 4.08 ± 0.33 | 3.78 ± 0.33 | 3.37 ± 0.33 | 3.57 ± 0.22^b^ |  |  |  |  |
|  | HIGH | 4.13 ± 0.33 | 3.42 ± 0.33 | 3.06 ± 0.33 | 3.24 ± 0.22^b^ |  |  |  |  |
|  |  |  |  |  |  |  |  |  |  |
| 22:4n6 | LOW | ND | 0.04 ± 0.02 | 0.00 ± 0.02 | 0.02 ± 0.01 |  | 0.38 | 0.33 | 0.38 |
|  | MOD | ND | 0.00 ± 0.02 | 0.00 ± 0.02 | ND |  |  |  |  |
|  | HIGH | ND | 0.00 ± 0.02 | 0.00 ± 0.02 | ND |  |  |  |  |
|  |  |  |  |  |  |  |  |  |  |
| 22:5n6 | LOW | ND | ND | ND | ND |  | - | - | - |
|  | MOD | ND | ND | ND | ND |  |  |  |  |
|  | HIGH | ND | ND | ND | ND |  |  |  |  |
|  |  |  |  |  |  |  |  |  |  |
| Total n-6 | LOW | 14.05 ± 1.13 | 16.28 ± 0.98 | 17.00 ± 0.98 | 16.64 ± 0.73^a^ |  | <0.01 | 0.34 | 0.99 |
|  | MOD | 12.77 ± 1.13 | 12.90 ± 0.99 | 13.54 ± 0.99 | 13.22 ± 0.74^b^ |  |  |  |  |
|  | HIGH | 14.15 ± 1.13 | 12.31 ± 0.98 | 13.16 ± 0.98 | 12.73 ± 0.74^b^ |  |  |  |  |
|  |  |  |  |  |  |  |  |  |  |
| 20:3n9 | LOW | ND | 0.00 ± 0.08 | 0.42 ± 0.08 | 0.21 ± 0.06 |  | 0.06 | <0.01 | 0.06 |
|  | MOD | ND | 0.00 ± 0.08 | 0.07 ± 0.08 | 0.04 ± 0.06 |  |  |  |  |
|  | HIGH | ND | 0.00 ± 0.08 | 0.06 ± 0.08 | 0.03 ± 0.06 |  |  |  |  |
|  |  |  |  |  |  |  |  |  |  |
| 18:3n3 (ALA) | LOW | 0.00 ± 0.00 | 0.22 ± 0.10 | 0.00 ± 0.10 | 0.11 ± 0.07^ab^ |  | 0.05 | <0.01 | 0.05 |
|  | MOD | 0.00 ± 0.00 | 0.09 ± 0.10 | 0.00 ± 0.10 | 0.04 ± 0.07^b^ |  |  |  |  |
|  | HIGH | 0.00 ± 0.00 | 0.60 ± 0.10 | 0.00 ± 0.10 | 0.30 ± 0.07^a^ |  |  |  |  |
|  |  |  |  |  |  |  |  |  |  |
| 20:3n3 | LOW | ND | ND | ND | ND |  | - | - | - |
|  | MOD | ND | ND | ND | ND |  |  |  |  |
|  | HIGH | ND | ND | ND | ND |  |  |  |  |
|  |  |  |  |  |  |  |  |  |  |
| 20:5n3 (EPA) | LOW | 0.19 ± 0.09 | 0.93 ± 0.20 | 0.21 ± 0.20 | 0.57 ± 0.16^b^ |  | <0.01 | 0.04 | 0.14 |
|  | MOD | 0.03 ± 0.09 | 1.91 ± 0.20 | 1.73 ± 0.20 | 1.82 ± 0.16^a^ |  |  |  |  |
|  | HIGH | 0.00 ± 0.09 | 2.11 ± 0.20 | 2.07 ± 0.20 | 2.09 ± 0.16^a^ |  |  |  |  |
|  |  |  |  |  |  |  |  |  |  |
| 22:5n3 | LOW | 0.49 ± 0.15 | 0.95 ± 0.22 | 0.80 ± 0.22 | 0.87 ± 0.15 |  | 0.46 | 0.77 | 0.83 |
|  | MOD | 0.29 ± 0.15 | 0.85 ± 0.22 | 0.72 ± 0.22 | 0.79 ± 0.15 |  |  |  |  |
|  | HIGH | 0.16 ± 0.15 | 0.55 ± 0.22 | 0.66 ± 0.22 | 0.61 ± 0.15 |  |  |  |  |
|  |  |  |  |  |  |  |  |  |  |
| 22:6n3 (DHA) | LOW | 1.03 ± 0.30 | 1.04 ± 0.22 | 0.84 ± 0.22 | 0.94 ± 0.16 |  | 0.23 | 0.97 | 0.62 |
|  | MOD | 0.49 ± 0.30 | 1.07 ± 0.22 | 1.29 ± 0.22 | 1.18 ± 0.16 |  |  |  |  |
|  | HIGH | 0.84 ± 0.30 | 1.35 ± 0.22 | 1.32 ± 0.22 | 1.33 ± 0.16 |  |  |  |  |
|  |  |  |  |  |  |  |  |  |  |
| EPA+DHA | LOW | 1.22 ± 0.35 | 1.96 ± 0.34 | 1.04 ± 0.34 | 1.50 ± 0.25^b^ |  | <0.01 | 0.23 | 0.28 |
|  | MOD | 0.52 ± 0.35 | 2.98 ± 0.34 | 3.01 ± 0.34 | 2.99 ± 0.25^a^ |  |  |  |  |
|  | HIGH | 0.84 ± 0.35 | 3.47 ± 0.33 | 3.40 ± 0.33 | 3.44 ± 0.24^a^ |  |  |  |  |
|  |  |  |  |  |  |  |  |  |  |
| Total n-3 | LOW | 1.71 ± 0.48 | 3.11 ± 0.46 | 1.82 ± 0.46 | 2.47 ± 0.32^b^ |  | <0.01 | 0.08 | 0.49 |
|  | MOD | 0.81 ± 0.48 | 3.92 ± 0.46 | 3.72 ± 0.46 | 3.82 ± 0.32^a^ |  |  |  |  |
|  | HIGH | 1.00 ± 0.48 | 4.64 ± 0.45 | 4.08 ± 0.45 | 4.36 ± 0.32^a^ |  |  |  |  |
|  |  |  |  |  |  |  |  |  |  |
| Total PUFA | LOW | 15.76 ± 1.53 | 19.22 ± 1.21 | 19.07 ± 1.21 | 19.15 ± 0.84 |  | 0.19 | 0.81 | 0.96 |
|  | MOD | 13.58 ± 1.53 | 16.84 ± 1.22 | 17.36 ± 1.22 | 17.10 ± 0.86 |  |  |  |  |
|  | HIGH | 15.15 ± 1.53 | 17.09 ± 1.21 | 17.44 ± 1.21 | 17.26 ± 0.84 |  |  |  |  |
|  |  |  |  |  |  |  |  |  |  |
| n-6: n-3 | LOW | 4.92 ± 2.03 | 6.06 ± 1.00 | 9.66 ± 1.19 | 7.86 ± 0.71^a^ |  | <0.01 | 0.08 | 0.52 |
|  | MOD | 4.20 ± 2.03 | 2.93 ± 1.20 | 4.35 ± 1.20 | 3.64 ± 0.73^b^ |  |  |  |  |
|  | HIGH | 6.66 ± 2.03 | 3.01 ± 1.09 | 3.93 ± 1.09 | 3.47 ± 0.69^b^ |  |  |  |  |
|  |  |  |  |  |  |  |  |  |  |
| AA: EPA+DHA | LOW | 2.43 ± 0.87 | 2.76 ± 0.49 | 4.25 ± 0.59 | 3.51 ± 0.34^a^ |  | <0.01 | <0.01 | <0.01 |
|  | MOD | 2.17 ± 0.87 | 1.37 ± 0.61 | 1.36 ± 0.61 | 1.37 ± 0.37^b^ |  |  |  |  |
|  | HIGH | 2.09 ± 0.87 | 1.02 ± 0.54 | 1.03 ± 0.54 | 1.02 ± 0.32^b^ |  |  |  |  |

^1^Mean indicates week data pooled across weeks

^a,b,c^Values in columns with different superscripts differ across time for a particular treatment group (*P* < 0.05)

**Supplementary Table S5.** Least squares means (LSMeans ± SEM) for serum fatty acids (%) in sphingomyelin (SM) of dogs (n = 27) across weeks (0, 4, and 8) and dose groups (DG) of eicosapentaenoic acid (EPA) and docosahexaenoic acid (DHA) [LOW (0.03 g/kg BW⁰·⁷⁵), MOD (0.45 g/kg BW⁰·⁷⁵), and HIGH (0.71 g/kg BW⁰·⁷⁵)] over 8 weeks, with corresponding p-values for main (DG, week) and interaction (DG × week) effects. Week 0 (pre-treatment baseline) was used as a covariate, and Mean values represent LSMeans averaged over Weeks 4 and 8 (baseline-adjusted). Different superscript letters (^a,b^) indicate statistically significant differences among DGs (P ≤ 0.05).

| Fatty acid in SM (%) |  | Week | | |  |  | P-value | | |
| --- | --- | --- | --- | --- | --- | --- | --- | --- | --- |
|  |  | 0 | 4 | 8 | Mean |  | DG | Week | DGxWeek |
| 14:0 | LOW | 1.02 ± 0.17 | 1.11 ± 0.19 | 0.89 ± 0.19 | 1.00 ± 0.11 |  | 0.36 | 0.76 | 0.39 |
|  | MOD | 1.23 ± 0.17 | 0.67 ± 0.19 | 0.97 ± 0.19 | 0.82 ± 0.11 |  |  |  |  |
|  | HIGH | 0.69 ± 0.17 | 0.93 ± 0.19 | 0.68 ± 0.19 | 0.81 ± 0.11 |  |  |  |  |
|  |  |  |  |  |  |  |  |  |  |
| 15:0 | LOW | 0.92 ± 0.29 | 0.48 ± 0.13 | 0.48 ± 0.13 | 0.48 ± 0.09 |  | 0.67 | 0.52 | 0.61 |
|  | MOD | 1.40 ± 0.29 | 0.49 ± 0.13 | 0.28 ± 0.13 | 0.38 ± 0.09 |  |  |  |  |
|  | HIGH | 0.78 ± 0.29 | 0.37 ± 0.13 | 0.39 ± 0.13 | 0.38 ± 0.09 |  |  |  |  |
|  |  |  |  |  |  |  |  |  |  |
| 16:0 | LOW | 31.48 ± 1.28 | 29.97 ± 1.73 | 31.61 ± 1.73 | 30.79 ± 1.38 |  | 0.74 | 0.46 | 0.82 |
|  | MOD | 29.90 ± 1.28 | 32.26 ± 1.74 | 32.12 ± 1.74 | 32.19 ± 1.38 |  |  |  |  |
|  | HIGH | 31.17 ± 1.28 | 30.31 ± 1.73 | 31.53 ± 1.73 | 30.92 ± 1.38 |  |  |  |  |
|  |  |  |  |  |  |  |  |  |  |
| 18:0 | LOW | 14.33 ± 0.46 | 19.40 ± 0.83 | 15.39 ± 0.83 | 17.40 ± 0.56 |  | 0.35 | <0.01 | 0.81 |
|  | MOD | 14.31 ± 0.46 | 18.51 ± 0.83 | 14.37 ± 0.83 | 16.44 ± 0.56 |  |  |  |  |
|  | HIGH | 14.94 ± 0.46 | 17.85 ± 0.84 | 14.75 ± 0.84 | 16.30 ± 0.57 |  |  |  |  |
|  |  |  |  |  |  |  |  |  |  |
| 19:0 | LOW | 2.17 ± 0.76 | 0.83 ± 0.17 | 1.10 ± 0.17 | 0.97 ± 0.13 |  | 0.85 | 0.60 | 0.17 |
|  | MOD | 3.09 ± 0.76 | 0.96 ± 0.17 | 0.78 ± 0.17 | 0.87 ± 0.13 |  |  |  |  |
|  | HIGH | 2.45 ± 0.76 | 1.03 ± 0.17 | 0.74 ± 0.17 | 0.89 ± 0.13 |  |  |  |  |
|  |  |  |  |  |  |  |  |  |  |
| 20:0 | LOW | 2.04 ± 0.25 | 1.14 ± 0.24 | 1.84 ± 0.24 | 1.49 ± 0.16 |  | 0.71 | 0.01 | 0.88 |
|  | MOD | 1.85 ± 0.25 | 1.39 ± 0.24 | 1.89 ± 0.24 | 1.64 ± 0.16 |  |  |  |  |
|  | HIGH | 2.05 ± 0.25 | 1.23 ± 0.24 | 1.70 ± 0.24 | 1.46 ± 0.16 |  |  |  |  |
|  |  |  |  |  |  |  |  |  |  |
| 22:0 | LOW | 2.79 ± 0.35 | 1.52 ± 0.41 | 2.26 ± 0.41 | 1.89 ± 0.26 |  | 0.20 | 0.01 | 0.66 |
|  | MOD | 2.57 ± 0.35 | 1.94 ± 0.41 | 3.00 ± 0.41 | 2.47 ± 0.26 |  |  |  |  |
|  | HIGH | 2.62 ± 0.35 | 1.75 ± 0.41 | 3.29 ± 0.41 | 2.52 ± 0.26 |  |  |  |  |
|  |  |  |  |  |  |  |  |  |  |
| 23:0 | LOW | 2.33 ± 0.27 | 1.41 ± 0.31 | 2.48 ± 0.31 | 1.95 ± 0.20 |  | 0.53 | <0.01 | 0.98 |
|  | MOD | 2.16 ± 0.27 | 1.76 ± 0.31 | 2.73 ± 0.31 | 2.24 ± 0.20 |  |  |  |  |
|  | HIGH | 2.23 ± 0.27 | 1.52 ± 0.31 | 2.46 ± 0.31 | 1.20 ± 0.20 |  |  |  |  |
|  |  |  |  |  |  |  |  |  |  |
| 24:0 | LOW | 2.56 ± 0.27 | 1.48 ± 0.34 | 2.74 ± 0.34 | 2.11 ± 0.23 |  | 0.41 | <0.01 | 0.97 |
|  | MOD | 2.73 ± 0.27 | 1.82 ± 0.34 | 3.21 ± 0.34 | 2.52 ± 0.23 |  |  |  |  |
|  | HIGH | 2.94 ± 0.27 | 1.85 ± 0.34 | 3.05 ± 0.34 | 2.45 ± 0.23 |  |  |  |  |
|  |  |  |  |  |  |  |  |  |  |
| Total SFA | LOW | 62.21 ± 1.95 | 57.46 ± 2.59 | 63.24 ± 2.59 | 60.35 ± 1.96 |  | 0.91 | 0.01 | 0.84 |
|  | MOD | 62.26 ± 1.95 | 59.46 ± 2.59 | 63.46 ± 2.59 | 61.46 ± 1.96 |  |  |  |  |
|  | HIGH | 61.78 ± 1.95 | 57.09 ± 2.59 | 63.87 ± 2.59 | 60.48 ± 1.96 |  |  |  |  |
|  |  |  |  |  |  |  |  |  |  |
| 16:1n7 | LOW | 0.11 ± 0.07 | 0.40 ± 0.09 | 0.30 ± 0.09 | 0.35 ± 0.07 |  | 0.48 | <0.01 | 0.39 |
|  | MOD | 0.07 ± 0.07 | 0.37 ± 0.09 | 0.16 ± 0.09 | 0.27 ± 0.07 |  |  |  |  |
|  | HIGH | 0.06 ± 0.07 | 0.55 ± 0.09 | 0.24 ± 0.09 | 0.39 ± 0.07 |  |  |  |  |
|  |  |  |  |  |  |  |  |  |  |
| 18:1n7 | LOW | 1.65 ± 0.21 | 1.97 ± 0.22 | 1.45 ± 0.22 | 1.71 ± 0.16 |  | 0.69 | <0.01 | 0.82 |
|  | MOD | 1.89 ± 0.21 | 2.00 ± 0.22 | 1.27 ± 0.22 | 1.63 ± 0.16 |  |  |  |  |
|  | HIGH | 1.68 ± 0.21 | 2.22 ± 0.22 | 1.43 ± 0.22 | 1.83 ± 0.16 |  |  |  |  |
|  |  |  |  |  |  |  |  |  |  |
| 18:1n9 | LOW | 4.45 ± 0.34 | 5.72 ± 0.32 | 4.59 ± 0.32 | 5.15 ± 0.22 |  | 0.30 | <0.01 | 0.96 |
|  | MOD | 4.69 ± 0.34 | 5.36 ± 0.32 | 4.21 ± 0.32 | 4.78 ± 0.22 |  |  |  |  |
|  | HIGH | 4.64 ± 0.34 | 5.75 ± 0.32 | 4.78 ± 0.32 | 5.26 ± 0.22 |  |  |  |  |
|  |  |  |  |  |  |  |  |  |  |
| 20:1n9 | LOW | 0.07 ± 0.04 | 0.07 ± 0.05 | 0.11 ± 0.05 | 0.09 ± 0.03 |  | 0.39 | 0.58 | 0.29 |
|  | MOD | 0.03 ± 0.04 | 0.07 ± 0.05 | 0.00 ± 0.05 | 0.03 ± 0.03 |  |  |  |  |
|  | HIGH | ND | 0.04 ± 0.05 | 0.14 ± 0.05 | 0.09 ± 0.03 |  |  |  |  |
|  |  |  |  |  |  |  |  |  |  |
| 22:1n9 | LOW | 6.40 ± 1.04 | 1.92 ± 0.37 | 3.31 ± 0.37 | 2.61 ± 0.27 |  | 0.31 | <0.01 | 0.10 |
|  | MOD | 7.50 ± 1.04 | 1.88 ± 0.37 | 4.52 ± 0.37 | 3.20 ± 0.27 |  |  |  |  |
|  | HIGH | 6.79 ± 1.04 | 2.49 ± 0.37 | 3.56 ± 0.37 | 3.03 ± 0.27 |  |  |  |  |
|  |  |  |  |  |  |  |  |  |  |
| 24:1n9 | LOW | 12.05 ± 1.30 | 5.14 ± 1.07 | 7.67 ± 1.07 | 6.41 ± 0.74 |  | 0.24 | 0.02 | 0.93 |
|  | MOD | 10.19 ± 1.30 | 7.02 ± 1.07 | 9.48 ± 1.07 | 8.25 ± 0.74 |  |  |  |  |
|  | HIGH | 10.70 ± 1.30 | 6.57 ± 1.06 | 8.33 ± 1.06 | 7.45 ± 0.73 |  |  |  |  |
|  |  |  |  |  |  |  |  |  |  |
| Total MUFA | LOW | 24.71 ± 1.10 | 15.26 ± 0.94 | 17.47 ± 0.94 | 16.36 ± 0.62 |  | 0.10 | 0.02 | 0.58 |
|  | MOD | 24.38 ± 1.10 | 16.67 ± 0.94 | 19.62 ± 0.94 | 18.14 ± 0.62 |  |  |  |  |
|  | HIGH | 23.87 ± 1.10 | 17.60 ± 0.94 | 18.46 ± 0.94 | 18.03 ± 0.62 |  |  |  |  |
|  |  |  |  |  |  |  |  |  |  |
| 18:2n6 (LA) | LOW | 8.26 ± 1.80 | 14.43 ± 1.90 | 10.67 ± 1.90 | 12.55 ± 1.29 |  | 0.38 | 0.03 | 0.97 |
|  | MOD | 8.14 ± 1.80 | 11.94 ± 1.90 | 7.99 ± 1.90 | 9.97 ± 1.29 |  |  |  |  |
|  | HIGH | 8.44 ± 1.80 | 12.90 ± 1.90 | 9.86 ± 1.90 | 11.38 ± 1.29 |  |  |  |  |
|  |  |  |  |  |  |  |  |  |  |
| 20:3n6 | LOW | 0.07 ± 0.10 | 0.69 ± 0.12 | 0.15 ± 0.12 | 0.42 ± 0.09 |  | 0.82 | <0.01 | 0.62 |
|  | MOD | 0.14 ± 0.10 | 0.76 ± 0.12 | -0.00 ± 0.12 | 0.38 ± 0.09 |  |  |  |  |
|  | HIGH | 0.20 ± 0.10 | 0.74 ± 0.12 | 0.18 ± 0.12 | 0.46 ± 0.09 |  |  |  |  |
|  |  |  |  |  |  |  |  |  |  |
| 20:4n6 (AA) | LOW | 2.45 ± 0.53 | 6.64 ± 1.09 | 4.08 ± 1.09 | 5.36 ± 0.86 |  | 0.24 | <0.01 | 0.99 |
|  | MOD | 2.45 ± 0.53 | 4.98 ± 1.09 | 2.68 ± 1.09 | 3.83 ± 0.86 |  |  |  |  |
|  | HIGH | 3.42 ± 0.53 | 4.52 ± 1.11 | 2.03 ± 1.11 | 3.27 ± 0.86 |  |  |  |  |
|  |  |  |  |  |  |  |  |  |  |
| 22:4n6 | LOW | ND | ND | ND | ND |  | - | - | - |
|  | MOD | ND | ND | ND | ND |  |  |  |  |
|  | HIGH | ND | ND | ND | ND |  |  |  |  |
|  |  |  |  |  |  |  |  |  |  |
| 22:5n6 | LOW | ND | ND | ND | ND |  | - | - | - |
|  | MOD | ND | ND | ND | ND |  |  |  |  |
|  | HIGH | ND | ND | ND | ND |  |  |  |  |
|  |  |  |  |  |  |  |  |  |  |
| Total n-6 | LOW | 11.10 ± 2.28 | 22.26 ± 2.93 | 15.32 ± 2.93 | 18.79 ± 2.15 |  | 0.35 | <0.01 | 0.99 |
|  | MOD | 11.49 ± 2.28 | 17.92 ± 2.93 | 11.12 ± 2.93 | 14.52 ± 2.14 |  |  |  |  |
|  | HIGH | 12.29 ± 2.28 | 18.55 ± 2.93 | 12.34 ± 2.93 | 15.44 ± 2.15 |  |  |  |  |
|  |  |  |  |  |  |  |  |  |  |
| 20:3n9 | LOW | ND | ND | ND | ND |  | - | - | - |
|  | MOD | ND | ND | ND | ND |  |  |  |  |
|  | HIGH | ND | ND | ND | ND |  |  |  |  |
|  |  |  |  |  |  |  |  |  |  |
| 18:3n3 (ALA) | LOW | 0.08 ± 0.10 | 0.35 ± 0.09 | 0.24 ± 0.09 | 0.29 ± 0.05 |  | 0.17 | 0.17 | 0.99 |
|  | MOD | 0.24 ± 0.10 | 0.23 ± 0.09 | 0.13 ± 0.09 | 0.18 ± 0.06 |  |  |  |  |
|  | HIGH | 0.04 ± 0.10 | 0.22 ± 0.09 | 0.09 ± 0.09 | 0.16 ± 0.05 |  |  |  |  |
|  |  |  |  |  |  |  |  |  |  |
| 20:3n3 | LOW | ND | ND | ND | ND |  | - | - | - |
|  | MOD | ND | ND | ND | ND |  |  |  |  |
|  | HIGH | ND | ND | ND | ND |  |  |  |  |
|  |  |  |  |  |  |  |  |  |  |
| 20:5n3 (EPA) | LOW | 0.05 ± 0.06 | 1.31 ± 0.36 | 0.31 ± 0.36 | 0.81 ± 0.27^b^ |  | <0.01 | <0.01 | 0.42 |
|  | MOD | 0.00 ± 0.06 | 1.69 ± 0.36 | 1.30 ± 0.36 | 1.50 ± 0.28^ab^ |  |  |  |  |
|  | HIGH | 0.15 ± 0.06 | 2.80 ± 0.37 | 1.56 ± 0.37 | 2.18 ± 0.28^a^ |  |  |  |  |
|  |  |  |  |  |  |  |  |  |  |
| 22:5n3 | LOW | 0.23 ± 0.10 | 1.01 ± 0.21 | 0.58 ± 0.21 | 0.80 ± 0.17 |  | 0.34 | <0.01 | 0.87 |
|  | MOD | 0.05 ± 0.10 | 0.76 ± 0.22 | 0.12 ± 0.22 | 0.44 ± 0.17 |  |  |  |  |
|  | HIGH | 0.15 ± 0.10 | 0.80 ± 0.21 | 0.28 ± 0.21 | 0.54 ± 0.17 |  |  |  |  |
|  |  |  |  |  |  |  |  |  |  |
| 22:6n3 (DHA) | LOW | 0.31 ± 0.16 | 1.13 ± 0.23 | 0.46 ± 0.23 | 0.80 ± 0.18 |  | 0.53 | <0.01 | 0.66 |
|  | MOD | 0.18 ± 0.16 | 1.23 ± 0.23 | 0.59 ± 0.23 | 0.91 ± 0.18 |  |  |  |  |
|  | HIGH | 0.46 ± 0.16 | 1.57 ± 0.23 | 0.60 ± 0.23 | 1.09 ± 0.18 |  |  |  |  |
|  |  |  |  |  |  |  |  |  |  |
| EPA+DHA | LOW | 0.36 ± 0.20 | 2.43 ± 0.54 | 0.77 ± 0.54 | 1.60 ± 0.41^b^ |  | 0.03 | <0.01 | 0.49 |
|  | MOD | 0.18 ± 0.20 | 2.87 ± 0.54 | 1.85 ± 0.54 | 2.36 ± 0.42^ab^ |  |  |  |  |
|  | HIGH | 0.62 ± 0.20 | 4.43 ± 0.55 | 2.23 ± 0.55 | 3.33 ± 0.42^a^ |  |  |  |  |
|  |  |  |  |  |  |  |  |  |  |
| Total n-3 | LOW | 0.67 ± 0.31 | 3.79 ± 0.69 | 1.57 ± 0.69 | 2.68 ± 0.54 |  | 0.22 | <0.01 | 0.68 |
|  | MOD | 0.47 ± 0.31 | 3.91 ± 0.70 | 2.14 ± 0.70 | 3.02 ± 0.54 |  |  |  |  |
|  | HIGH | 0.81 ± 0.31 | 5.42 ± 0.70 | 2.56 ± 0.70 | 3.99 ± 0.54 |  |  |  |  |
|  |  |  |  |  |  |  |  |  |  |
| Total PUFA | LOW | 11.77 ± 2.49 | 26.02 ± 3.43 | 16.86 ± 3.43 | 21.44 ± 2.59 |  | 0.59 | <0.01 | 0.99 |
|  | MOD | 11.96 ± 2.49 | 21.89 ± 3.43 | 13.33 ± 3.43 | 17.61 ± 2.59 |  |  |  |  |
|  | HIGH | 13.09 ± 2.49 | 23.93 ± 3.43 | 14.86 ± 3.43 | 19.40 ± 2.60 |  |  |  |  |
|  |  |  |  |  |  |  |  |  |  |
| n-6: n-3 | LOW | 3.32 ± 2.05 | 6.92 ± 1.24 | 11.02 ± 1.32 | 9.46 ± 0.86^a^ |  | 0.01 | 0.08 | 0.18 |
|  | MOD | 5.66 ± 2.05 | 4.63 ± 1.17 | 5.43 ± 1.17 | 7.60 ± 0.75^ab^ |  |  |  |  |
|  | HIGH | 3.56 ± 2.05 | 3.91 ± 1.17 | 4.71 ± 1.32 | 5.57 ± 0.78^b^ |  |  |  |  |
|  |  |  |  |  |  |  |  |  |  |
| AA: EPA+DHA | LOW | 1.21 ± 0.61 | 3.24 ± 0.48 | 4.16 ± 0.68 | 3.56 ± 0.41^a^ |  | 0.05 | 0.63 | 0.20 |
|  | MOD | 0.84 ± 0.61 | 1.68 ± 0.46 | 1.34 ± 0.46 | 2.28 ± 0.42^ab^ |  |  |  |  |
|  | HIGH | 1.27 ± 0.61 | 1.03 ± 0.46 | 1.01 ± 0.51 | 1.61 ± 0.37^b^ |  |  |  |  |

^1^Mean indicates week data pooled across weeks

^a,b,c^Values in columns with different superscripts differ across time for a particular treatment group (*P* < 0.05)

**Supplementary Table S6.** Least squares means (LSMeans ± SEM) for total fatty acid (FA) content (ul/ml serum) of dogs (n = 27) across weeks (0, 4, and 8) and dose groups (DG) of eicosapentaenoic acid (EPA) and docosahexaenoic acid (DHA) [LOW (0.03 g/kg BW⁰·⁷⁵), MOD (0.45 g/kg BW⁰·⁷⁵), and HIGH (0.71 g/kg BW⁰·⁷⁵)] over 8 weeks, with corresponding p-values for main (DG, week) and interaction (DG × week) effects. Week 0 (pre-treatment baseline) was used as a covariate, and Mean values represent LSMeans averaged over Weeks 4 and 8 (baseline-adjusted). Different superscript letters (^a,b^) indicate statistically significant differences among DGs (P ≤ 0.05).

| Total FA (ul/ml serum) | | Week | | | |  | P-value | | |
| --- | --- | --- | --- | --- | --- | --- | --- | --- | --- |
|  |  | 0 | 4 | 8 | Mean |  | DG | Week | DGxWeek |
| 14:0 | LOW | 2.03 ± 0.18 | 1.56 ± 0.40 | 2.79 ± 0.36 | 2.15 ± 0.20 |  | 0.26 | <0.01 | .0.29 |
|  | MOD | 1.61 ± 0.24 | 0.93 ± 0.07 | 2.02 ± 0.23 | 1.58 ± 0.13 |  |  |  |  |
|  | HIGH | 1.99 ± 0.13 | 1.99 ± 0.65 | 2.17 ± 0.17 | 2.05 ± 0.22 |  |  |  |  |
|  |  |  |  |  |  |  |  |  |  |
| 15:0 | LOW | 1.25 ± 0.11 | 1.03 ± 0.23 | 1.66 ± 0.13 | 1.32 ± 0.10 |  | 0.40 | <0.01 | 0.17 |
|  | MOD | 0.97 ± 0.14 | 0.63 ± 0.13 | 1.48 ± 0.14 | 1.06 ± 0.09 |  |  |  |  |
|  | HIGH | 1.18 ± 0.05 | 1.37 ± 0.41 | 1.40 ± 0.14 | 1.32 ± 0.14 |  |  |  |  |
|  |  |  |  |  |  |  |  |  |  |
| 16:0 | LOW | 123.54 ± 11.70 | 104.28 ± 31.32 | 166.35 ± 16.23 | 132.43 ± 12.34^a^ |  | 0.06 | <0.01 | 0.34 |
|  | MOD | 106.99 ± 16.70 | 48.93 ± 9.46 | 118.81 ±11.98 | 95.54 ± 8.42^b^ |  |  |  |  |
|  | HIGH | 117.73 ± 7.03 | 105.76 ± 30.95 | 121.50 ± 10.55 | 115.00 ± 10.79^ab^ |  |  |  |  |
|  |  |  |  |  |  |  |  |  |  |
| 18:0 | LOW | 122.72 ± 13.52 | 107.37 ± 34.99 | 162.94 ± 15.89 | 131.92 ± 13.13 |  | 0.14 | <0.01 | 0.40 |
|  | MOD | 112.46 ± 18.37 | 55.00 ± 11.01 | 126.62 ± 11.48 | 102.19 ± 8.73 |  |  |  |  |
|  | HIGH | 117.26 ± 9.56 | 120.88 ± 36.38 | 135.27 ± 13.78 | 124.47 ± 12.92 |  |  |  |  |
|  |  |  |  |  |  |  |  |  |  |
| 19:0 | LOW | 1.59 ± 0.15 | 1.39 ± 0.26 | 1.68 ± 0.14 | 1.56 ± 0.10 |  | 0.39 | 0.04 | 0.22 |
|  | MOD | 1.30 ± 0.16 | 0.93 ± 0.19 | 1.99 ± 0.27 | 1.45 ± 0.12 |  |  |  |  |
|  | HIGH | 1.42 ± 0.14 | 1.73 ± 0.43 | 1.87 ± 0.26 | 1.67 ± 0.17 |  |  |  |  |
|  |  |  |  |  |  |  |  |  |  |
| 20:0 | LOW | 1.15 ± 0.10 | 1.06 ± 0.33 | 1.39 ± 0.18 | 1.21 ± 0.12 |  | 0.19 | 0.05 | 0.57 |
|  | MOD | 0.96 ± 0.14 | 0.54 ± 0.12 | 1.26 ± 0.16 | 0.95 ± 0.08 |  |  |  |  |
|  | HIGH | 1.10 ± 0.10 | 1.26 ± 0.44 | 1.48 ± 0.18 | 1.28 ± 0.16 |  |  |  |  |
|  |  |  |  |  |  |  |  |  |  |
| 22:0 | LOW | 3.72 ± 0.77 | 3.91 ± 0.36 | 3.91 ± 0.36 | 3.39 ± 0.39 |  | 0.38 | 0.36 | 0.12 |
|  | MOD | 2.95 ± 0.43 | 1.26 ± 0.27 | 3.46 ± 0.61 | 2.67 ± 0.29 |  |  |  |  |
|  | HIGH | 3.54 ± 0.47 | 4.54 ± 1.99 | 2.99 ± 0.28 | 3.69 ± 0.67 |  |  |  |  |
|  |  |  |  |  |  |  |  |  |  |
| 23:0 | LOW | 0.99 ± 0.10 | 1.10 ± 0.47 | 0.92 ± 0.12 | 1.00 ± 0.15 |  | 0.55 | 0.88 | 0.26 |
|  | MOD | 0.99 ± 0.15 | 0.59 ± 0.23 | 1.16 ± 0.11 | 0.95 ± 0.08 |  |  |  |  |
|  | HIGH | 1.11 ± 0.22 | 1.35 ± 0.50 | 1.06 ± 0.16 | 1.17 ± 0.18 |  |  |  |  |
|  |  |  |  |  |  |  |  |  |  |
| 24:0 | LOW | 1.61 ± 0.22 | 1.26 ± 0.46 | 1.33 ± 0.13 | 1.41 ± 0.16 |  | 0.63 | 0.67 | 0.42 |
|  | MOD | 1.53 ± 0.40 | 0.79 ± 0.33 | 1.32 ± 0.19 | 1.27 ± 0.16 |  |  |  |  |
|  | HIGH | 1.26 ± 0.11 | 1.46 ± 0.48 | 1.19 ± 0.14 | 1.30 ± 0.17 |  |  |  |  |
|  |  |  |  |  |  |  |  |  |  |
| Total SFA | LOW | 258.60 ± 25.87 | 288.60 ± 69.13 | 342.95 ± 32.36 | 276.39 ± 26.30 |  | 0.10 | <0.01 | 0.36 |
|  | MOD | 229.74 ± 36.36 | 108.86 ± 5.02 | 258.12 ± 24.12 | 207.66 ± 17.78 |  |  |  |  |
|  | HIGH | 246.58 ± 16.76 | 240.34 ± 72.01 | 268.93 ± 25.13 | 251.95 ± 25.13 |  |  |  |  |
|  |  |  |  |  |  |  |  |  |  |
| 16:1n7 | LOW | 9.02 ± 1.06 | 7.48 ± 1.96 | 13.15 ± 1.86 | 9.98 ± 1.01 |  | 0.17 | <0.01 | 0.17 |
|  | MOD | 7.07 ± 1.09 | 4.14 ± 0.84 | 9.17 ± 1.09 | 7.05 ± 0.60 |  |  |  |  |
|  | HIGH | 9.60 ± 0.87 | 8.38 ± 2.32 | 9.05 ± 0.67 | 9.01 ± 0.83 |  |  |  |  |
|  |  |  |  |  |  |  |  |  |  |
| 18:1c9 | LOW | 95.54 ± 9.33 | 73.85 ± 21.95 | 131.94 ± 15.70 | 101.47 ± 9.91^a^ |  | 0.07 | <0.01 | 0.26 |
|  | MOD | 79.09 ± 12.71 | 35.53 ± 7.32 | 91.22 ± 10.83 | 71.54 ± 6.79^b^ |  |  |  |  |
|  | HIGH | 91.50 ± 4.50 | 75.87 ± 20.25 | 92.36 ± 8.26 | 86.58 ± 7.30^b^ |  |  |  |  |
|  |  |  |  |  |  |  |  |  |  |
| 18:1c11 | LOW | 19.65 ± 2.07 | 15.77 ± 5.04 | 22.95 ± 2.19 | 19.60 ± 1.86 |  | 0.14 | 0.03 | 0.30 |
|  | MOD | 17.26 ± 2.96 | 8.17 ± 1.60 | 17.11 ± 1.66 | 14.82 ± 1.31 |  |  |  |  |
|  | HIGH | 19.68 ± 1.48 | 17.24 ± 5.03 | 17.49 ± 1.56 | 18.14 ± 1.77 |  |  |  |  |
|  |  |  |  |  |  |  |  |  |  |
| 20:3n9 | LOW | 1.28 ± 0.10 | 0.98 ± 0.28 | 2.23 ± 0.26 | 1.52 ± 0.16^a^ |  | <0.01 | <0.01 | <0.01 |
|  | MOD | 1.20 ± 0.19 | 0.36 ± 0.11 | 1.05 ± 0.13 | 0.92 ± 0.10^b^ |  |  |  |  |
|  | HIGH | 1.25 ± 0.13 | 0.73 ± 0.22 | 0.85 ± 0.11 | 0.94 ± 0.10^b^ |  |  |  |  |
|  |  |  |  |  |  |  |  |  |  |
| 22:1n9 | LOW | 9.58 ± 3.89 | 9.06 ± 3.37 | 8.76 ± 0.32 | 9.14 ± 1.63 |  | 0.32 | 0.58 | 0.38 |
|  | MOD | 5.27 ± 0.79 | 5.41 ± 0.94 | 8.46 ± 0.26 | 6.57 ± 0.33 |  |  |  |  |
|  | HIGH | 5.95 ± 0.19 | 17.63 ± 10.31 | 8.53 ± 0.43 | 10.70 ± 3.45 |  |  |  |  |
|  |  |  |  |  |  |  |  |  |  |
| 24:1n9 | LOW | 4.19 ± 0.41 | 3.35 ± 1.38 | 2.33 ± 0.25 | 3.29 ± 0.46 |  | 0.43 | 0.23 | 0.31 |
|  | MOD | 3.54 ± 0.59 | 1.82 ± 0.43 | 2.35 ± 0.18 | 2.70 ± 0.24 |  |  |  |  |
|  | HIGH | 3.94 ± 0.35 | 4.00 ± 1.34 | 2.19 ± 0.23 | 3.38 ± 0.48 |  |  |  |  |
|  |  |  |  |  |  |  |  |  |  |
| Total MUFA | LOW | 139.26 ± 12.58 | 148.60 ± 33.88 | 181.36 ± 19.72 | 144.98 ± 13.72 |  | 0.15 | 0.01 | 0.25 |
|  | MOD | 113.43 ± 18.00 | 55.37 ± 2.60 | 129.36 ± 13.71 | 103.60 ± 9.04 |  |  |  |  |
|  | HIGH | 131.91 ± 6.48 | 123.85 ± 38.54 | 130.48 ± 10.83 | 128.74 ± 13.00 |  |  |  |  |
|  |  |  |  |  |  |  |  |  |  |
| 18:2n6 (LA) | LOW | 211.68 ± 22.68 | 178.69 ± 54.96 | 294.80 ± 32.51 | 230.30 ± 22.73^a^ |  | 0.02 | <0.01 | 0.39 |
|  | MOD | 186.43 ± 32.23 | 72.24 ± 13.92 | 192.84 ± 21.18 | 157.41 ± 15.91^b^ |  |  |  |  |
|  | HIGH | 199.93 ± 14.82 | 159.61 ± 47.06 | 198.01 ± 21.39 | 185.85 ± 17.60^ab^ |  |  |  |  |
|  |  |  |  |  |  |  |  |  |  |
| 20:3n6 | LOW | 8.13 ± 1.22 | 6.16 ± 1.58 | 9.96 ± 1.38 | 8.16 ± 0.81 |  | 0.10 | <0.01 | 0.22 |
|  | MOD | 5.97 ± 0.89 | 2.74 ± 0.85 | 6.93 ± 0.80 | 5.44 ± 0.50 |  |  |  |  |
|  | HIGH | 6.94 ± 0.92 | 5.98 ± 1.81 | 6.45 ± 0.74 | 6.46 ± 0.70 |  |  |  |  |
|  |  |  |  |  |  |  |  |  |  |
| 20:4n6 (AA) | LOW | 102.59 ± 11.32 | 121.72 ± 30.77 | 144.82 ± 15.37 | 114.17 ± 11.81^a^ |  | 0.02 | <0.01 | 0.28 |
|  | MOD | 97.08 ± 16.76 | 37.30 ± 7.10 | 88.12 ± 8.78 | 77.76 ± 7.74^b^ |  |  |  |  |
|  | HIGH | 99.66 ± 8.42 | 99.66 ± 8.42 | 83.28 ± 7.20 | 86.63 ± 8.71^b^ |  |  |  |  |
|  |  |  |  |  |  |  |  |  |  |
| 22:4n6 | LOW | 3.60 ± 0.30 | 3.37 ± 0.78 | 7.35 ± 0.82 | 4.83 ± 0.51^a^ |  | <0.01 | <0.01 | <0.01 |
|  | MOD | 3.14 ± 0.47 | 1.08 ± 0.46 | 2.72 ± 0.33 | 2.43 ± 0.25^b^ |  |  |  |  |
|  | HIGH | 3.35 ± 0.31 | 1.87 ± 0.53 | 2.38 ± 0.26 | 2.53 ± 0.24^b^ |  |  |  |  |
|  |  |  |  |  |  |  |  |  |  |
| 22:5n6 | LOW | 1.47 ± 0.14 | 1.38 ± 0.35 | 2.24 ± 0.39 | 1.71 ± 0.19^a^ |  | <0.01 | 0.31 | 0.18 |
|  | MOD | 1.51 ± 0.46 | 0.93 ± 0.37 | 1.08 ± 0.11 | 1.23 ± 0.19^b^ |  |  |  |  |
|  | HIGH | 1.37 ± 0.13 | 1.22 ± 0.35 | 0.96 ± 0.11 | 1.18 ± 0.13^b^ |  |  |  |  |
|  |  |  |  |  |  |  |  |  |  |
| Total n-6 | LOW | 327.46 ± 35.03 | 375.15 ± 88.22 | 459.18 ± 47.55 | 359.16 ± 35.41^a^ |  | 0.02 | <0.01 | 0.33 |
|  | MOD | 294.13 ± 50.09 | 113.00 ± 6.21 | 291.69 ± 30.69 | 244.26 ± 24.21^b^ |  |  |  |  |
|  | HIGH | 311.26 ± 23.73 | 245.62 ± 73.83 | 291.07 ± 29.44 | 282.65 ± 27.11^b^ |  |  |  |  |
|  |  |  |  |  |  |  |  |  |  |
| 20:3n9 | LOW | 1.28 ± 0.10 | 0.98 ± 0.28 | 2.23 ± 0.26 | 1.52 ± 0.16^a^ |  | <0.01 | <0.01 | <0.01 |
|  | MOD | 1.20 ± 0.19 | 0.36 ± 0.11 | 1.05 ± 0.13 | 0.92 ± 0.10^b^ |  |  |  |  |
|  | HIGH | 1.25 ± 0.13 | 0.73 ± 0.22 | 0.85 ± 0.11 | 0.94 ± 0.10^b^ |  |  |  |  |
|  |  |  |  |  |  |  |  |  |  |
| 18:3n3 (ALA) | LOW | 4.14± 0.37 | 3.59 ± 1.13 | 6.95 ± 0.99 | 4.94 ± 0.56^a^ |  | 0.06 | <0.01 | 0.42 |
|  | MOD | 3.54 ± 0.59 | 1.66 ± 0.30 | 5.25 ± 0.71 | 3.61 ± 0.40^b^ |  |  |  |  |
|  | HIGH | 3.56 ± 0.24 | 3.21 ± 0.84 | 4.89 ± 0.37 | 3.89 ± 0.34^b^ |  |  |  |  |
|  |  |  |  |  |  |  |  |  |  |
| 18:4n3 (SDA) | LOW | 0.61 ± 0.13 | 0.37 ± 0.14 | 1.00 ± 0.23 | 0.67 ± 0.11 |  | 0.33 | 0.01 | 0.54 |
|  | MOD | 0.62 ± 0.14 | 0.12 ± 0.07 | 0.60 ± 0.16 | 0.47 ± 0.08 |  |  |  |  |
|  | HIGH | 0.53 ± 0.10 | 0.50 ± 0.35 | 0.70 ± 0.19 | 0.58 ± 0.13 |  |  |  |  |
|  |  |  |  |  |  |  |  |  |  |
| 20:5n3 (EPA) | LOW | 5.87 ± 0.94 | 8.03 ± 3.64 | 6.15 ± 0.51 | 6.63 ± 1.14^b^ |  | <0.01 | 0.01 | 0.12 |
|  | MOD | 5.15 ± 0.76 | 19.43 ± 3.67 | 47.84 ± 4.85 | 24.33 ± 3.81^ab^ |  |  |  |  |
|  | HIGH | 5.44 ± 0.57 | 45.03 ± 11.72 | 67.63 ± 10.72 | 67.63 ± 10.72^a^ |  |  |  |  |
|  |  |  |  |  |  |  |  |  |  |
| 22:5n3 | LOW | 8.15 ± 0.89 | 6.87 ± 1.80 | 10.54 ± 1.29 | 8.59 ± 0.79 |  | 0.19 | <0.01 | 0.22 |
|  | MOD | 7.23 ± 1.04 | 4.60 ± 1.10 | 13.92 ± 1.48 | 8.85 ± 0.92 |  |  |  |  |
|  | HIGH | 7.04 ± 0.68 | 9.10 ± 2.15 | 14.07 ± 2.22 | 10.07 ± 1.17 |  |  |  |  |
|  |  |  |  |  |  |  |  |  |  |
| 22:6n3 (DHA) | LOW | 13.43 ± 1.74 | 10.34 ± 3.31 | 11.13 ± 1.23 | 11.68 ± 1.22^b^ |  | <0.01 | 0.01 | 0.14 |
|  | MOD | 11.25 ± 1.77 | 9.81 ± 2.44 | 22.35 ± 2.04 | 14.88 ± 1.33^b^ |  |  |  |  |
|  | HIGH | 11.96 ± 1.03 | 20.54 ± 4.89 | 25.77 ± 2.85 | 19.42 ± 2.15^a^ |  |  |  |  |
|  |  |  |  |  |  |  |  |  |  |
| EPA+DHA | LOW | 19.29 ± 2.66 | 22.06 ± 6.92 | 17.28 ± 1.67 | 18.31 ± 2.28^b^ |  | <0.01 | 0.01 | 0.13 |
|  | MOD | 16.40 ± 2.43 | 29.85 ± 2.01 | 70.18 ± 6.23 | 39.21 ± 4.89^ab^ |  |  |  |  |
|  | HIGH | 17.40 ± 1.57 | 65.57 ± 16.59 | 93.40 ± 13.48 | 58.79 ± 9.22^a^ |  |  |  |  |
|  |  |  |  |  |  |  |  |  |  |
| Total n-3 | LOW | 32.19 ± 3.68 | 36.26 ± 9.88 | 35.77 ± 3.44 | 32.51 ± 3.37^b^ |  | <0.01 | <0.01 | 0.18 |
|  | MOD | 27.78 ± 4.11 | 36.19 ± 2.16 | 89.95 ± 7.58 | 52.15 ± 5.89^b^ |  |  |  |  |
|  | HIGH | 28.52 ± 2.46 | 78.38 ± 19.74 | 113.07 ± 15.70 | 73.32 ± 10.59^a^ |  |  |  |  |
|  |  |  |  |  |  |  |  |  |  |
| Total PUFA | LOW | 359.65 ± 38.44 | 411.41 ± 98.01 | 494.94 ± 50.70 | 391.67 ± 38.54 |  | 0.10 | <.01 | 0.45 |
|  | MOD | 321.91 ± 54.07 | 149.19 ± 7.93 | 381.64 ± 36.14 | 296.42 ± 27.02 |  |  |  |  |
|  | HIGH | 339.78 ± 26.10 | 324.00 ± 93.23 | 404.14 ± 43.72 | 355.97 ± 34.69 |  |  |  |  |
|  |  |  |  |  |  |  |  |  |  |
| n-6:n-3 | LOW | 10.24 ± 0.44 | 10.28 ± 0.65 | 12.91 ± 0.64 | 11.08 ± 0.41^a^ |  | <0.01 | <0.63 | 0.34 |
|  | MOD | 9.43 ± 1.17 | 3.15 ± 0.63 | 3.27 ± 0.25 | 5.66 ± 0.69^b^ |  |  |  |  |
|  | HIGH | 11.02 ± 0.27 | 3.06 ± 0.19 | 2.70 ± 0.19 | 5.60 ± 0.76^b^ |  |  |  |  |

| AA: EPA + DHA | LOW | 5.45 ± 0.33 | 5.58 ± 0.54 | 8.38 ± 0.42 | 6.44 ± 0.37^a^ |  | <0.01 | <0.01 | 0.27 |
| --- | --- | --- | --- | --- | --- | --- | --- | --- | --- |
|  | MOD | 5.26 ± 0.66 | 1.27 ± 0.13 | 1.28 ± 0.13 | 2.81 ± 0.43^b^ |  |  |  |  |
|  | HIGH | 5.80 ± 0.23 | 1.12 ± 0.07 | 0.95 ± 0.07 | 2.61 ± 0.45^b^ |  |  |  |  |

^1^Mean indicates week data pooled across weeks

^a,b,c^Values in columns with different superscripts differ across time for a particular treatment group (*P* < 0.05)
